# PRMT1-Mediated PARP1 Methylation Drives Lung Metastasis and Chemoresistance via P65 Activation in Triple-Negative Breast Cancer

**DOI:** 10.34133/research.0854

**Published:** 2025-09-08

**Authors:** Jinhui Zhang, Zirui Huang, Cailu Song, Song Wu, Jindong Xie, Yutian Zou, Xiaoming Xie, Tao Wu, Han Yang, Hailin Tang

**Affiliations:** ^1^ State Key Laboratory of Oncology in South China, Guangdong Provincial Clinical Research Center for Cancer, Sun Yat-sen University Cancer Center, Guangzhou 510060, China.; ^2^ Changde Hospital, Xiangya School of Medicine, Central South University (The First People’s Hospital of Changde City), Changde, China.

## Abstract

Triple-negative breast cancer (TNBC) is the most aggressive breast cancer subtype, characterized by a high propensity for metastasis, poor prognosis, and limited treatment options. Research has demonstrated a substantial correlation between the expression of protein arginine N-methyltransferase 1 (PRMT1) and enhanced proliferation, metastasis, and poor outcomes in TNBC. However, the specific role of PRMT1 in lung metastasis and chemoresistance remains unclear. Single-cell RNA sequencing coupled with bioinformatics analysis was employed to identify pertinent genes within metastatic TNBC samples. Functional assays, including cell cycle, apoptosis, wound healing, Transwell migration, colony formation, and Cell Counting Kit-8 Assay (CCK-8), were conducted to evaluate the role of PRMT1. The interaction between PRMT1 and PARP1 was validated by mass spectrometry (MS) and immunoprecipitation. Downstream signaling pathways were explored, with a focus on P65 activation. Enzyme-linked immunosorbent assay was used to quantify the effect of PRMT1 on interleukin-1β secretion. Our study identified a significant association between elevated PRMT1 expression and both lung metastasis and chemoresistance in TNBC. PRMT1 boosts TNBC cell growth, invasion, and lung metastasis. Additionally, high PRMT1 expression contributed to increased resistance to docetaxel in TNBC. Mechanistically, PRMT1 methylates PARP1. On the one hand, this methylation promotes the DNA damage repair ability of PAPA1. On the other hand, it in turn modulates the NF-κB signaling pathway. This modulation enhances the stemness of tumor cells and induces immune suppression within the tumor microenvironment, thereby exacerbating chemoresistance in TNBC. PRMT1 drives lung metastasis and chemoresistance in TNBC through PARP1 methylation and P65 activation. These findings position PRMT1 as a promising biomarker and therapeutic target to overcome resistance and limit metastatic progression in TNBC.

## Introduction

Breast cancer is characterized by malignant tumors arising from the transformation of epithelial tissue within the breast. It ranks among the most prevalent forms of cancer globally, with an incidence rate surpassed only by lung cancer [1]. Data from 2022 indicate that breast cancer has emerged as the fifth leading cause of cancer-related mortality worldwide, accounting for 665,684 deaths, a figure that significantly exceeds that of other gynecological cancers. Concurrently, approximately 2.31 million women globally received a new diagnosis of breast cancer, underscoring a continuous global increase in incidence, with an annual growth rate of approximately 3.1% [2]. In China, official statistics reveal that breast cancer has become the most prevalent malignancy among women, with incidence rates escalating annually. The rate of increase in China is double the global average, positioning the country as having the highest breast cancer incidence worldwide [3]. Approximately 20% to 30% of patients experience local recurrence or distant metastasis within 2 years following diagnosis [4,5]. For those with distant metastasis, the 5-year survival rate falls below 25% [6,7]. Nearly 90% of cancer-related deaths is closely associated with metastatic spread. Among the various organs, the lungs are the most frequent site of metastasis [8,9]. Once metastasis occurs, the median survival time typically ranges from 6 to 18 months [10]. Triple-negative breast cancer (TNBC) has a poor prognosis and few treatment options [11,12]. Furthermore, patients with TNBC may develop resistance to therapeutic interventions during treatment, thereby elevating the risk of recurrence and metastasis, which complicates the therapeutic landscape [13,14]. In TNBC, tumor stem cells are crucial in both the initiation and progression of the disease [15]. Tumor stem cells have the ability to self-renew and differentiate into various cell types, contributing to the heterogeneity of the tumor and its aggressive behavior. These cells are often resistant to conventional therapies, including chemotherapy, which leads to tumor recurrence and metastasis [16,17]. The presence of tumor stem cells in TNBC is a key factor in chemoresistance, as they are less sensitive to the cytotoxic effects of many chemotherapeutic agents [18,19]. Additionally, the immune microenvironment in TNBC plays a significant role in the chemoresistance [20]. The tumor-associated immune cells, such as tumor-associated macrophages, myeloid-derived suppressor cells, and regulatory T cells, contribute to immune evasion and tumor progression. These cells often suppress the antitumor immune response, allowing the tumor to grow unchecked and resist treatments [21]. The immune microenvironment can also influence the function and survival of tumor stem cells, further exacerbating chemoresistance [22]. Consequently, comprehensive investigations into the molecular mechanisms underlying distant metastasis and chemoresistance in TNBC, alongside the identification of effective therapeutic targets, have emerged as pressing clinical issues necessitating attention in contemporary medical research [23,24].

Protein arginine N-methyltransferase 1 (PRMT1) is a pivotal enzyme involved in the regulation of protein arginine methylation, a crucial posttranslational modification [25]. This methylation process, particularly targeting arginine residues, is fundamental in modulating a diverse array of cellular functions, including cellular signaling, gene expression, and protein interactions [26]. Through the modification of arginine residues in substrate proteins, PRMT1 influences protein stability, subcellular localization, and interaction dynamics [27]. Furthermore, its role in apoptosis, or programmed cell death, is vital for the removal of damaged or superfluous cells, thereby preventing the accumulation of dysfunctional cells that could contribute to pathologies such as cancer [28]. The impact of PRMT1 on tumorigenesis highlights its potential as a therapeutic target, underscoring its significance in both physiological and pathological contexts [29]. PRMT1’s ability to influence oncogenic pathways makes it a key target for cancer treatments, especially in breast cancer, where it affects metastasis and drug resistance [30]. Additionally, its role in regulating immune responses highlights its importance beyond cancer [31]. PRMT1 significantly contributes to chemoresistance in TNBC by altering the methylation of key molecules like epidermal growth factor receptor (EGFR) and phosphoglycerate dehydrogenase (PHGDH), which strengthens tumor resilience [32,33]. Understanding how PRMT1 induces drug resistance could lead to new strategies to overcome it and improve current treatments. Overall, PRMT1 is a key enzyme crucial for biological functions, cancer progression, immune regulation, and chemoresistance [34].

NF-κB signaling pathway contributes to various facets of tumor progression, including cell proliferation, immune evasion, and resistance to apoptosis, thereby rendering it a viable target for therapeutic interventions [35,36]. Notably, NF-κB signaling pathway exerts substantial influence in the development and metastasis of TNBC, a subtype characterized by its aggressive behavior and lack of targeted therapies [37]. In TNBC cells, NF-κB signaling pathway is frequently hyperactivated, fostering an environment conducive to tumor growth [38]. This abnormal activation helps cancer cells survive and multiply by altering gene expression linked to cell survival [39]. It modulates the expression of various molecules integral to cellular invasion, including matrix metalloproteinases [40]. Through the promotion of tumor cell invasion, NF-κB signaling pathway expedites the metastatic cascade, allowing the cancer to disseminate to distant organs, which markedly deteriorates the prognosis for TNBC patients [41]. The release of pro-inflammatory cytokines exacerbates the tumor’s aggressive phenotype and enhances its ability to evade immune surveillance [42,43]. Moreover, the involvement of NF-κB signaling pathway in conferring resistance to chemotherapy presents a significant challenge in the treatment of TNBC [44,45]. While many chemotherapeutic agents exert their effects by inducing apoptosis in tumor cells, the NF-κB signaling pathway contributes to drug resistance by controlling the expression of genes like MDR1 [46]. The continuous activation of the NF-κB signaling pathway enhances TNBC cell survival and treatment resistance, complicating its clinical management and highlighting its crucial role in TNBC pathogenesis [47,48].

We aim to clarify the link between PRMT1 expression and lung metastasis progression in TNBC. To achieve this, we integrated single-cell RNA sequencing technology with bioinformatics analysis to systematically examine the gene expression profiles of metastatic breast cancer tissues. Through comprehensive molecular analyses and experimental validation, we showed that PRMT1 greatly increases TNBC cell proliferation and migration. This effect was particularly evident during lung metastasis, where PRMT1 activity appears to be crucial in facilitating the dissemination and colonization of cancer cells in the pulmonary environment. Through the NF-κB signaling pathway, PRMT1 enhances the metastatic potential of TNBC cells, facilitating their survival and proliferation in distant organs, such as the lungs. In this context, PRMT1 acts as a catalytic agent, facilitating and amplifying these processes, thereby promoting the metastatic dissemination of TNBC. Importantly, this finding has clinical implications, as it indicates that PRMT1 may contribute to chemoresistance. Our research highlights the pivotal role of PRMT1 in the lung metastasis of TNBC, as well as its involvement in chemoresistance. Consequently, PRMT1 emerges as a promising candidate for further investigation. By targeting PRMT1, it may be possible to develop innovative treatment strategies that not only inhibit metastatic spread but also overcome drug resistance in TNBC.

## Results

### Elevated PRMT1 levels are linked to lung metastasis and chemoresistance in TNBC

**Fig. 1.**
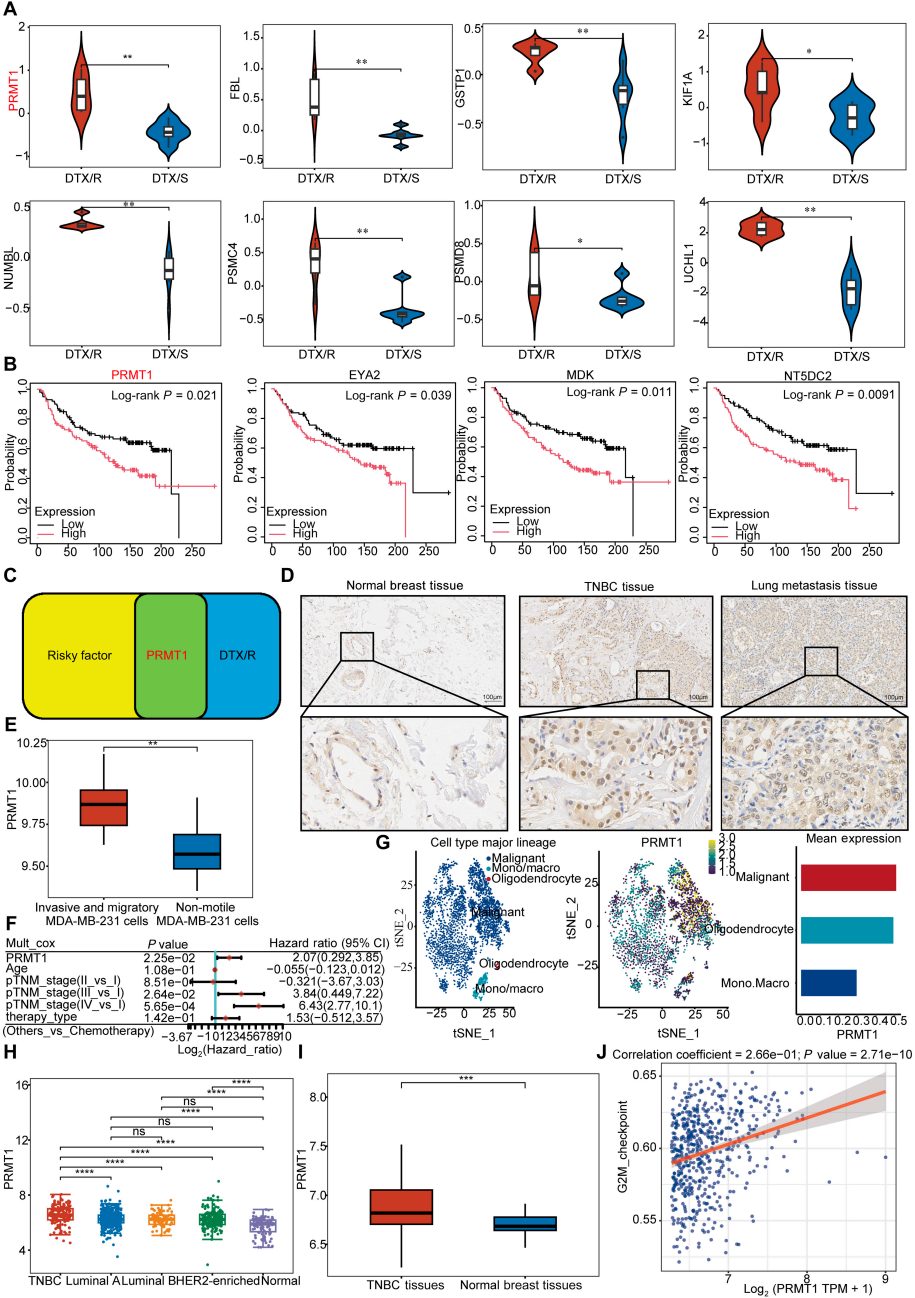
Elevated PRMT1 levels are linked to lung metastasis and chemoresistance in TNBC. (A) Differential analysis was performed using the GSE110153 dataset. (B) Survival analysis was conducted by utilizing the GSE7390 dataset. (C) The Venn diagram demonstrates the intersection of up-regulated genes in resistant breast cancer tissues with genes identified as risk factors. (D) Representative images display PRMT1 immunohistochemical staining in breast cancer tumor tissues, corresponding normal tissues, and lung metastatic tissues. (E) A box plot illustrates the differences in PRMT1 expression between metastatic and nonmetastatic MDA-MB-231 cells in the GSE54465 dataset. (F) Multivariate Cox analyses provide the *P* values, hazard ratios (HR), and confidence intervals (CI) for PRMT1 expression and clinical characteristics. (G) The t-distributed stochastic neighbor embedding (t-SNE) plot depicts PRMT1 expression levels within the breast cancer single-cell RNA sequencing cohort BRCAGSE143423. (H) Differential expression analysis of PRMT1 was performed using TCGA database. (I) A box plot compares PRMT1 expression levels in the GSE76250 dataset. (J) A Spearman correlation analysis plot was conducted to examine the role of PRMT1 in the G_2_/M checkpoint.

We performed single-cell RNA sequencing on metastatic lesions from 3 breast cancer patients, including one who had received docetaxel prior to metastasis. Notably, PRMT1—a gene linked to cell cycle regulation—was consistently overexpressed across all samples, suggesting an important role in both metastasis and chemoresistance in breast cancer. To substantiate this observation, we utilized the GSE110153 dataset and differential expression analysis of this dataset demonstrated a significant up-regulation of PRMT1 expression in the tissues of chemoresistance patients (Fig. 1A). Additionally, we performed survival analysis using the GSE7390 dataset, which includes data from patients with distant metastatic breast cancer. Upon analyzing this dataset, patients with elevated PRMT1 levels tended to exhibit lower survival rates following treatment, and this association was even more pronounced in those who developed metastasis (Fig. 1B). PRMT1 is crucial for breast cancer’s metastasis and chemoresistance, with its expression level closely linked to patient outcomes (Fig. 1C). PRMT1 levels were elevated in MDA-MB-231 and BT-549 cells compared to MCF-10A cells (Fig. S1B and C). Using The Cancer Genome Atlas (TCGA) for pan-cancer analysis, we found that PRMT1 expression is significantly higher in breast cancer tumors than in normal tissue (Fig. S1E). Immunohistochemical staining revealed increased PRMT1 expression in breast cancer tissues compared to normal tissue, with even higher levels in lung metastases than in primary tumors (Fig. 1D). We utilized data from the GSE76250 dataset, which comprises expression profiles of TNBC and corresponding normal breast tissue, alongside the GSE54465 dataset, which includes expression profiles of metastatic and nonmetastatic MDA-MB-231 cells. PRMT1 expression is further elevated in metastatic TNBC cells relative to nonmetastatic TNBC cells (Fig. 1E and I). Multivariate Cox analysis showed a significant link between PRMT1 and tumor-node-metastasis (TNM) staging in TNBC. Then, based on the results of the multivariate Cox proportional hazards model, we constructed a Nomogram using the “rms” package to predict the total recurrence rate for 3 years (Fig. 1F and Fig. S1F). We investigated the expression levels of PRMT1 within the single-cell RNA sequencing cohorts BRCA-GSE143423 and BRCA-GSE150660, and the analysis of these datasets demonstrated that PRMT1 expression is predominantly localized in the malignant epithelial cells of breast cancer (Fig. 1G and Fig. S1A). Data from TCGA were utilized to examine the differential expression of PRMT1 across various breast cancer tissue types and adjacent normal breast tissues. The findings revealed that PRMT1 expression is most elevated in TNBC tissues (Fig. 1H). A Spearman correlation analysis plot revealed a positive link between PRMT1 expression and the G_2_/M checkpoint (Fig. 1J).

### PRMT1 promotes the proliferation and migration of TNBC

**Fig. 2.**
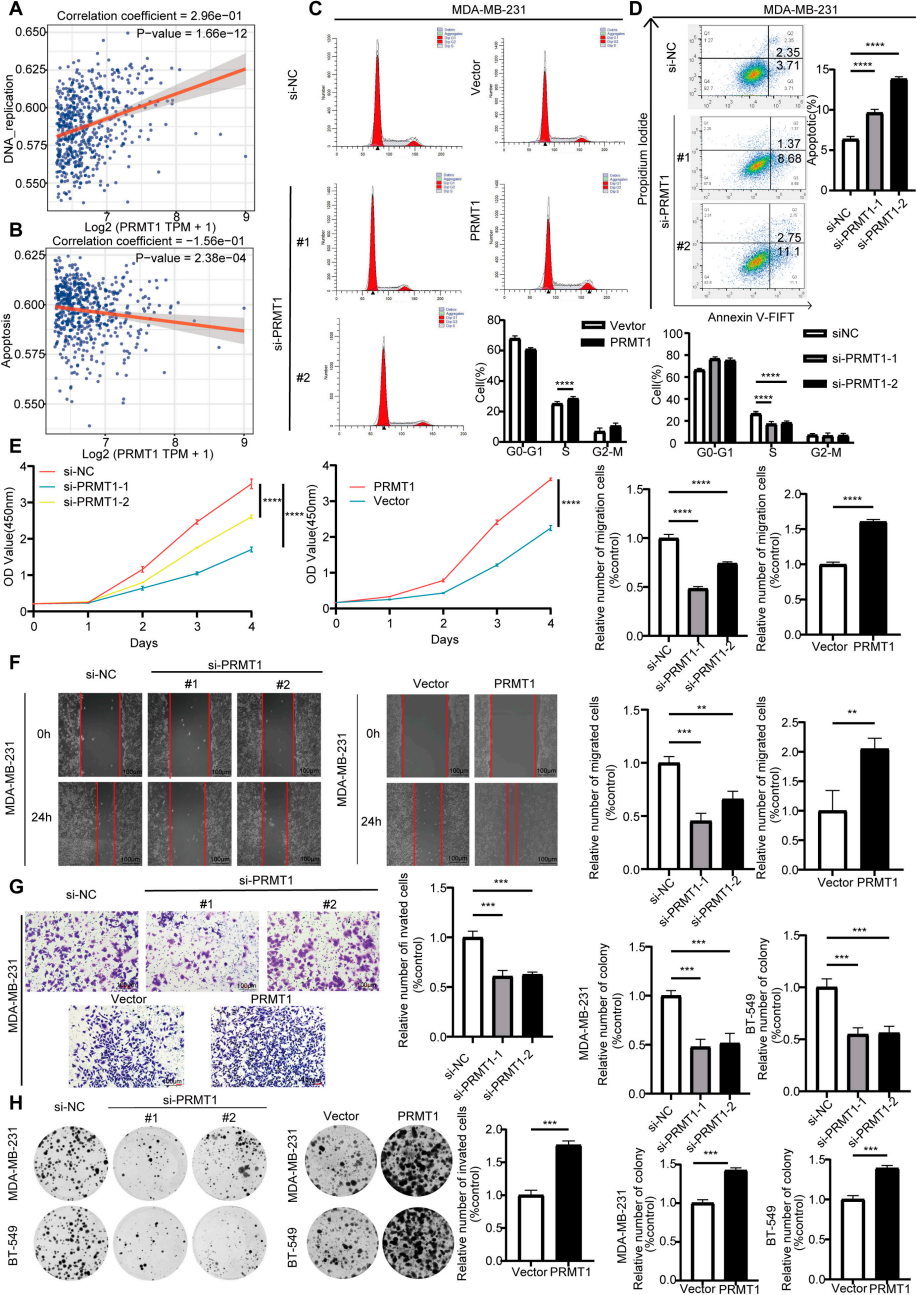
PRMT1 promotes the proliferation and migration of TNBC. (A) Within the TCGA database, single-sample gene set enrichment analysis (ssGSEA) was performed to assess the expression levels of PRMT1 and its association with DNA replication. (B) Within the TCGA database, ssGSEA was performed to assess the expression levels of PRMT1 and its association with apoptosis. (C) Flow cytometry was utilized to analyze the cell cycle distribution of MDA-MB-231 cells following the knockdown and overexpression of PRMT1. (D) Flow cytometry was utilized to examine the apoptosis of MDA-MB-231 cells subsequent to the knockdown of PRMT1. (E) The CCK-8 assay was employed to determine the growth curve of MDA-MB-231 cells following the knockdown and overexpression of PRMT1. (F) The wound healing assay was employed to assess the migratory capacity of MDA-MB-231 cells following the knockdown and overexpression of PRMT1. (G) The Transwell migration assay was conducted to evaluate the invasive capacity of MDA-MB-231 cells subsequent to the knockdown and overexpression of PRMT1. (H) The colony formation assay was conducted to evaluate the colony-forming potential of MDA-MB-231 and BT-549 cells, subsequent to the knockdown and overexpression of PRMT1.

PRMT1 expression was positively correlated with DNA replication and negatively correlated with apoptosis pathways in TNBC, highlighting its potential role in promoting tumor cell survival and proliferation (Fig. 2A and B). Flow cytometry analysis demonstrated that silencing PRMT1 resulted in a decreased proportion of cells in the S phase. Conversely, overexpression of PRMT1 led to a significant increase in the proportion of cells in the S phase (Fig. 2C and Fig. S2C). Furthermore, apoptosis assays indicated that PRMT1 knockdown elevated the apoptosis rate (Fig. 2D and Fig. S2D). In the CCK-8 assay, the down-regulation of PRMT1 expression was associated with a reduced proliferation rate in TNBC. Conversely, PRMT1 overexpression enhanced the proliferation of TNBC (Fig. 2E and Fig. S2E). In the wound healing assay, inhibiting PRMT1 slowed wound closure in TNBC, while overexpressing PRMT1 accelerated it (Fig. 2F and Fig. S2G). In the Transwell migration, inhibiting PRMT1 significantly reduced the migration of TNBC cells, while overexpressing PRMT1 increased their migration (Fig. 2G and Fig. S2F). Colony formation assays demonstrated that PRMT1 knockdown significantly diminished colony formation in TNBC, whereas PRMT1 overexpression led to an increase in colony numbers (Fig. 2H). Collectively, these experimental results underscore the role of PRMT1 as a facilitator of the migration and invasion capabilities of TNBC.

### PRMT1 contributes to docetaxel resistance in TNBC, while TC-E 5003 can inhibit TNBC cell growth and migration

**Fig. 3.**
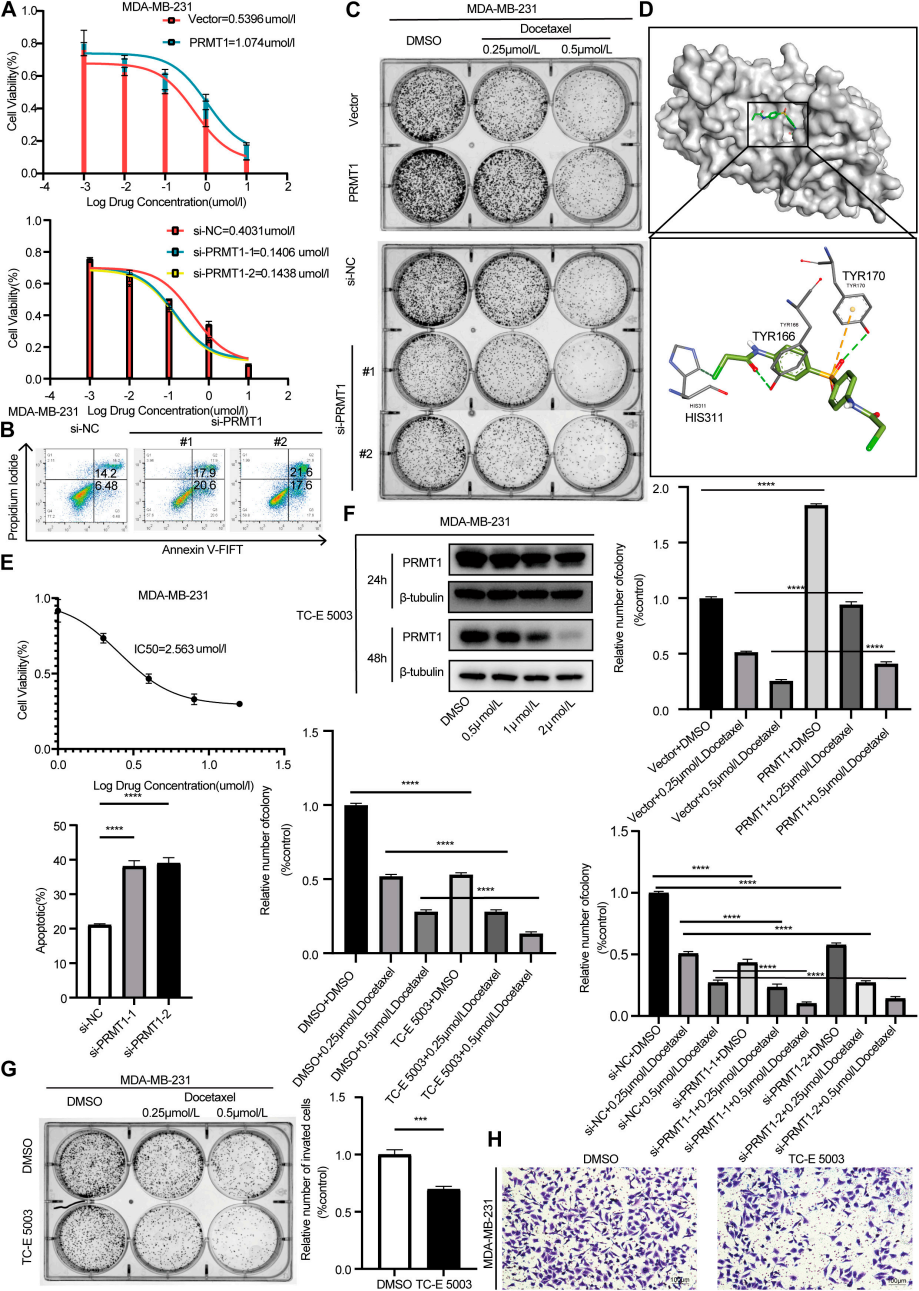
PRMT1 contributes to docetaxel resistance in TNBC, while TC-E 5003 can inhibit TNBC cell growth and migration. (A) Evaluate the IC_50_ values of MDA-MB-231 cells transfected with either a PRMT1 overexpression plasmid or siRNA. (B) Treat MDA-MB-231 cells transfected with PRMT1 siRNA with either docetaxel or dimethyl sulfoxide, and subsequently quantify the apoptosis rate via flow cytometry. (C) The colony formation ability of MDA-MB-231 cells with PRMT1 knockdown and overexpression was evaluated by a colony formation assay after treatment with docetaxel. (D) Analyze the 3D structure of the interaction between the PRMT1 protein and the small-molecule TC-E 5003. (E) Ascertain the IC_50_ value of TC-E 5003 in MDA-MB-231 cells. (F) Investigate the expression levels of PRMT1 in MDA-MB-231 cells following treatment with varying concentrations and time intervals of TC-E 5003. (G) The colony formation assay was employed to assess the effects of combined treatment with TC-E 5003 and docetaxel on the MDA-MB-231 cells. (H) Utilize a Transwell migration assay to assess the invasive potential of MDA-MB-231 cells after exposure to TC-E 5003.

To elucidate the role of PRMT1 in docetaxel resistance, we assessed the median inhibitory concentration (IC₅₀) values of MDA-MB-231 and BT-549 cells following PRMT1 overexpression or knockdown via small interfering RNA (siRNA). The results demonstrated that cells transfected with the PRMT1 overexpression plasmids exhibited higher IC₅₀ values compared to the control group upon docetaxel treatment. In contrast, PRMT1 siRNA-transfected MDA-MB-231 and BT-549 cells showed lower IC₅₀ values compared to the control group under identical treatment conditions (Fig. 3A and Fig. S3A). Furthermore, flow cytometry analysis indicated a significant increase in the apoptosis rate of PRMT1 knockdown cells treated with docetaxel, as compared to the control group (Fig. 3B and Fig. S3B). In the colony formation assay, it was observed that the colony-forming ability of PRMT1 knockdown cells treated with docetaxel was diminished compared to the control group, whereas PRMT1 overexpression in docetaxel-treated cells resulted in enhanced colony formation (Fig. 3C and Fig. S3C). TC-E 5003, a selective PRMT1 inhibitor, is recognized for its anti-inflammatory effects, effectively modulating lipopolysaccharide (LPS)-induced AP-1 and NF-κB signaling pathway to reduce inflammation [49,50]. Molecular docking experiments indicated that TC-E 5003 can form a stable complex with PRMT1 (Fig. 3D and Fig. S3D). IC₅₀ assays demonstrated that TC-E 5003 effectively inhibited the growth of TNBC cell lines (Fig. 3E and Fig. S3E). Western blot revealed that PRMT1 expression exhibited both concentration-dependent and time-dependent alterations under varying concentrations and durations of TC-E 5003 treatment (Fig. 3F and Fig. S3F). It also reduced cell invasion and migration, with fewer cells crossing the membrane in the Transwell migration assay (Fig. 3G and Fig. S3G) More importantly, the synergistic application of TC-E 5003 and docetaxel demonstrates a more pronounced efficacy in inhibiting the proliferation of TNBC cells (Fig. 3H and Fig. S3H).

### Arginine methylation mediated by PRMT1 enhances the activation of PARP1

**Fig. 4.**
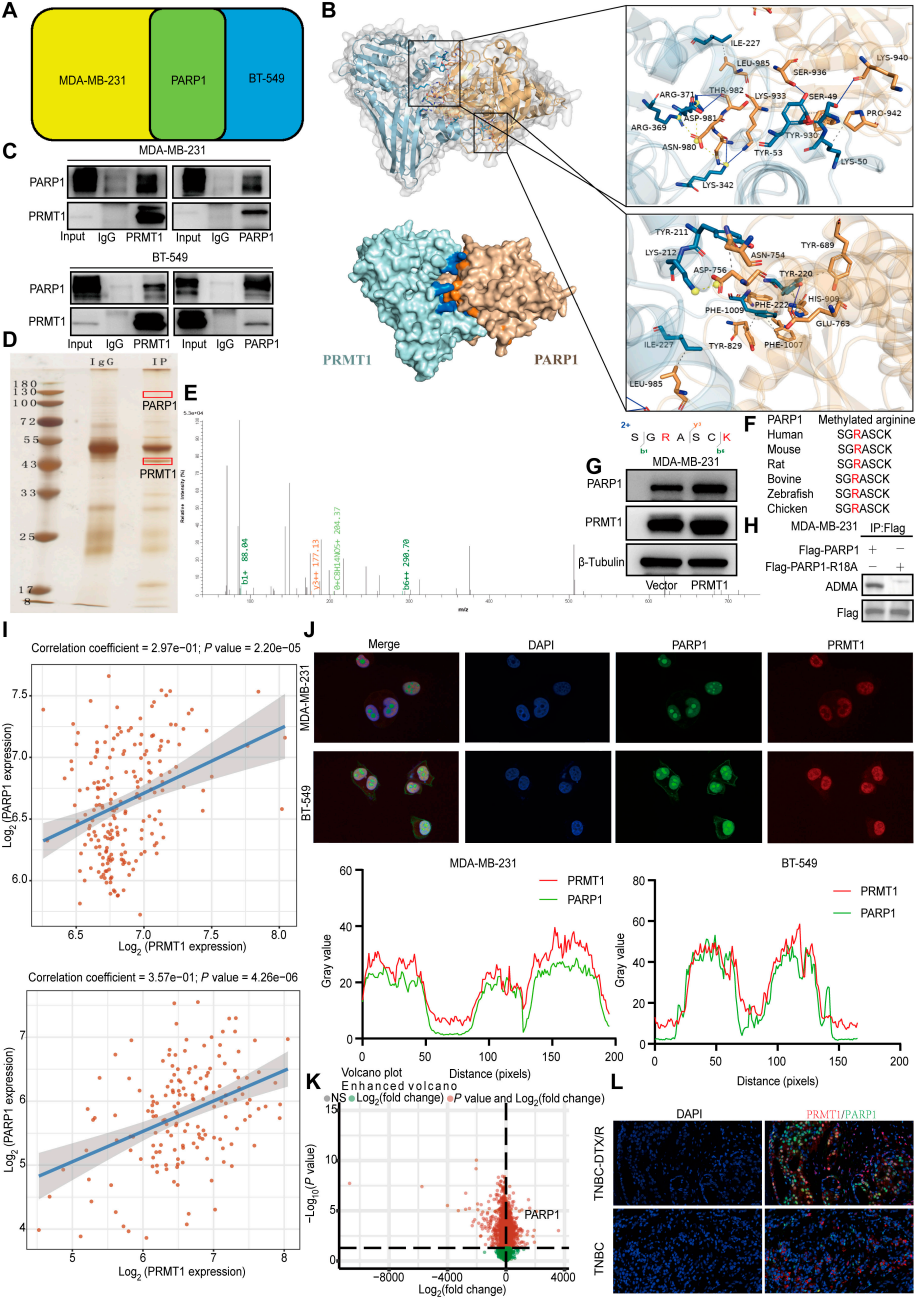
Arginine methylation mediated by PRMT1 enhances the activation of PARP1. (A) MS analysis indicated that PARP1 functions as a downstream target of PRMT1. (B) The molecular docking of PRMT1 and PARP1 was used to observe the binding. (C and D) Immunoprecipitation experiment and silver staining assay were employed to investigate the interaction between PRMT1 and PARP1. (E and F) The methylation sites of PARP1 were identified using MS. (G) Expression correlation between PRMT1 and PARP1 in MDA-MB-231 cells. (H) The methylation sites of PARP1 were verified by immunoprecipitation. (I) Gene–gene correlation between PRMT1 and PARP1 was conducted using data from the TCGA database and the GSE76250 dataset. (J) Immunofluorescence staining was utilized to evaluate the expression levels of PRMT1 and PARP1 in MDA-MB-231 and BT-549 cell. (K) The expression of PARP1 in chemoresistant TNBC tissues was assessed using the GSE28784 dataset. (L) Immunofluorescence was used to compare the expression of PRMT1 and PARP1 in docetaxel-resistant breast cancer tissues relative to TNBC tissues.

Utilizing MS analysis, we identified PARP1 as a potential downstream target of PRMT1 (Fig. 4A). Through molecular docking experiments, we found that PRMT1 can be stably collected with PARP1 (Fig. 4B).This interaction was validated by immunoprecipitation, confirming that PRMT1 physically associates with PARP1 and supports its role as a downstream effector (Fig. 4C). Furthermore, silver staining of proteins provided additional evidence that PRMT1 may mediate its biological effects within cells through its interaction with PARP1 (Fig. 4D). Further analysis of the methylated modification sites by MS analysis revealed that the 18th site of PARP1 undergoes asymmetric dimethylation (Fig. 4E). Additionally, the sequences targeted by PRMT1 are typically highly conserved, and the sequence of PARP1 methylation sites analyzed by MS also exhibits high conservation (Fig. 4F). Western blot revealed that PRMT1 overexpression significantly increased PARP1 expression (Fig. 4G and Fig. S4A). Through further immunoprecipitation experiments with mutants, we confirmed that PARP1 amino acid 18 can be asymmetric arginine dimethylation (Fig. 4H and Fig. S4D). Gene correlation analysis using the TCGA database and GSE76250 dataset showed a positive correlation between PRMT1 and PARP1 expression (Fig. 4I). Through analysis of the TCGA database, both PRMT1 and PARP1 are highly expressed in TNBC (Fig. S4B). Protein–protein interaction (PPI) analysis revealed that PRMT1 interacts with PARP1 (Fig. S4C). Immunofluorescence experiments further demonstrated colocalization of PRMT1 and PARP1 fluorescence signals within the cell nucleus, implying that these proteins may collaboratively function in the nuclear environment (Fig. 4J). The expression of PARP1 was high in metastatic TNBC cells using the GSE54465 dataset (Fig. S4E). An analysis of the GSE28784 dataset revealed an up-regulation of PARP1 expression in docetaxel-resistant breast cancer tissues (Fig. 4K). In docetaxel-resistant breast cancer tissues, the expression of PRMT1 and PARP1 was increased (Fig. 4L). Furthermore, PRMT1 was implicated in DNA damage repair mechanisms within TNBC, and in the TCGA database and GSE76250 dataset, PRMT1 was positively correlated with DNA damage repair enzyme expression (Fig. S4F, G, and H).

### PRMT1 facilitates the proliferation and lung metastasis of TNBC in vivo

**Fig. 5.**
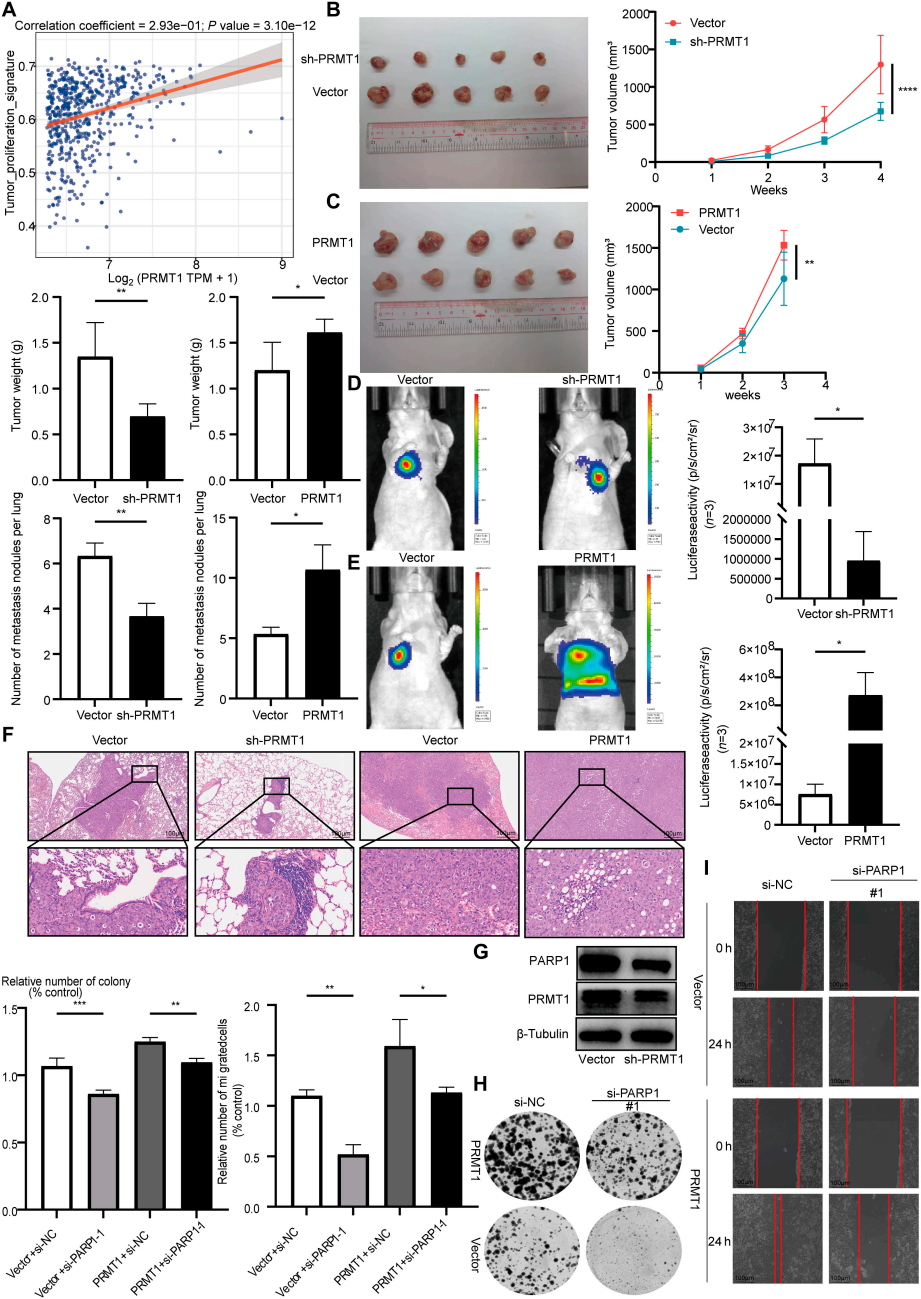
PRMT1 facilitates the proliferation and lung metastasis of TNBC in vivo. (A) A Spearman correlation analysis plot was conducted to investigate the relationship between PRMT1 and tumor progression. (B and C) A subcutaneous breast cancer injection model was developed to assess the carcinogenic potential of PRMT1. Tumor volume was measured weekly, and growth curves were plotted for each experimental group. Subsequently, tumor weight was recorded. (D and E) A TNBC lung metastasis model was established to evaluate the role of PRMT1 in the lung metastasis of TNBC. The impact of PRMT1 on the metastatic potential of TNBC was analyzed using bioluminescence imaging. (F) H&E staining was employed to evaluate tumor tissue metastasis in the lung metastasis model groups. (G) Western blot was used to determine the expression levels of PRMT1 and PARP1 in sh-PRMT1 cells. (H and I) A wound healing assay and a colony formation assay were conducted to assess functional rescue.

A Spearman correlation analysis plot indicates that PRMT1 expression is linked to tumor progression (Fig. 5A). Upon injection of cells with overexpressed PRMT1 into nude mice, tumor growth rate was notably accelerated, culminating in increased tumor volume and weight. Conversely, injection of PRMT1 knockdown cells into nude mice resulted in tumors that were smaller than those in the control group. Tumors in the PRMT1 knockdown cohort demonstrated reduced volumes and weights, accompanied by slower growth rates (Fig. 5B and C). To further elucidate the role of PRMT1 in lung metastasis of TNBC, a lung metastasis model was developed via tail vein injection. The findings indicated that PRMT1 overexpression led to an increased number of metastatic lesions in the lungs and an accelerated metastasis rate. Conversely, PRMT1 knockdown slowed metastasis and reduced lung lesions (Fig. 5D and E). We used hematoxylin and eosin (H&E) staining to examine PRMT1’s role in lung metastasis of TNBC. It found that PRMT1 overexpression increased the number and size of lung metastases; conversely, reducing PRMT1 decreased and dispersed lung metastases (Fig. 5F). To further investigate the relationship between PRMT1 and PARP1, Western blot was used to determine the expression levels of PRMT1 and PARP1 in sh-PRMT1 cells and a functional complementation experiment was performed by silencing PARP1 in PRMT1-overexpressing cells (Fig. 5G to I).

### PRMT1 facilitates the activation of the NF-κB signaling pathway through the regulation of PARP1

**Fig. 6.**
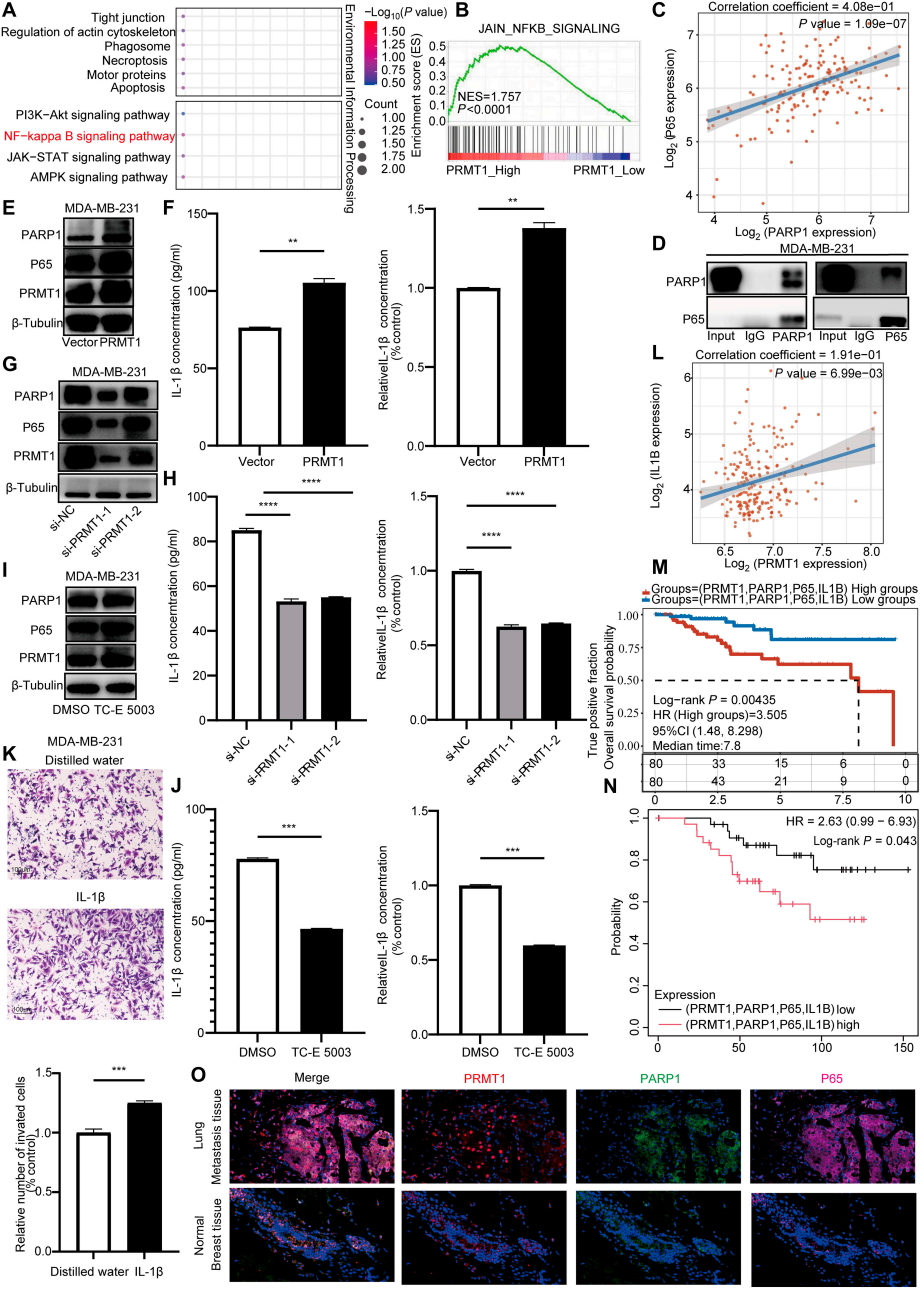
PRMT1 facilitates the activation of the NF-κB signaling pathway through the regulation of PARP1. (A) Perform KEGG pathway enrichment analysis on the proteins identified by MS. (B) Perform pathway enrichment analysis of PRMT1 using the TCGA database. (C and D) Conduct gene–gene correlation of PARP1 and P65 in the TCGA database, and validate the findings through immunoprecipitation experiments. (E and F) Analyze the expression levels of P65 and IL-1β of MDA-MB-231 cells after PRMT1 overexpression using Western blot analysis and ELISA assays. (G and H) Analyze the expression levels of P65 and IL-1β of MDA-MB-231 cells after PRMT1 knockdown using Western blot analysis and ELISA assays. (I and J) The expression levels of P65 and IL-1β of MDA-MB-231 cells after TC-E 5003 treatment were analyzed using Western blot analysis and ELISA assays. (K) A Transwell migration assay was used to analyze the invasive ability of MDA-MB-231 cells after treatment with recombinant IL-1β protein. (L) The correlation between PRMT1 and IL1B was verified in the TCGA database. (M and N) A prognostic model was constructed through LASSO Cox regression analysis of the PRMT1, PARP1, P65, and IL1B genes and was validated in the dataset GSE6532. (O) The expression levels of PRMT1, PARP1, and P65 in TNBC lung metastasis tissue and normal breast tissue were determined using multiplex immunofluorescence.

MS-based Kyoto Encyclopedia of Genes and Genomes (KEGG) pathway enrichment analysis revealed significant involvement of the NF-κB signaling pathway among the enriched proteins (Fig. 6A). To corroborate this observation, we conducted pathway enrichment analysis utilizing the TCGA database, which demonstrated a strong association of both PRMT1 and PARP1 with the NF-κB signaling pathway (Fig. 6B and Fig. S6A). Analysis of datasets GSE66495 and GSE43502 revealed that the NF-κB signaling pathway and cytokines are activated during breast cancer metastasis (Fig. S6B and C). The NF-κB signaling pathway and cytokines are also activated in the chemoresistant breast cancer tissues (Fig. S5D and E). Research indicates that PARP1 boosts P65’s transcriptional activity, activating the NF-κB signaling pathway and regulating interleukin-1β (IL-1β) expression and inflammation [51,52]. Gene–gene correlation shows a positive link between PARP1 and P65 levels (Fig. 6C). Immunoprecipitation experiments also confirmed PARP1’s interaction with P65 (Fig. 6D and Fig. S6E). Knocking down PARP1 reduces P65 and IL-1β levels (Fig. S6F and G). PRMT1 has been shown to influence the expression of IL-1β (Fig. 6L and Fig. S6H). PRMT1 overexpression increases PARP1, P65, and IL-1β in TNBC (Fig. 6E and F and Fig. S6I and K), while PRMT1 knockdown decreases them (Fig. 6G and H and Fig. S6J and L). TC-E 5003 treatment also lowers PARP1, P65, and IL-1β levels (Fig. 6I and J and Fig. S6M and N). By adding recombinant IL-1β protein, we found that IL-1β can promote invasion and metastasis in TNBC (Fig. 6K and Fig. S6O). A LASSO Cox regression model incorporating PRMT1, PARP1, P65, and IL-1B (Fig. 6M) indicates that these molecules are linked to poor prognosis in breast cancer, confirmed by the GSE6532 dataset (Fig. 6N). Using multiplex immunofluorescence staining, we found higher levels of PRMT1, PARP1, and P65 in lung metastatic tissues of TNBC compared to normal breast tissues (Fig. 6O). This suggests that PRMT1 up-regulation may promote these molecules’ expression, potentially increasing metastasis and chemoresistance via the NF-κB signaling pathway.

### PRMT1 enhances tumor stemness and immune suppression in TNBC, sustaining chemoresistance

**Fig. 7.**
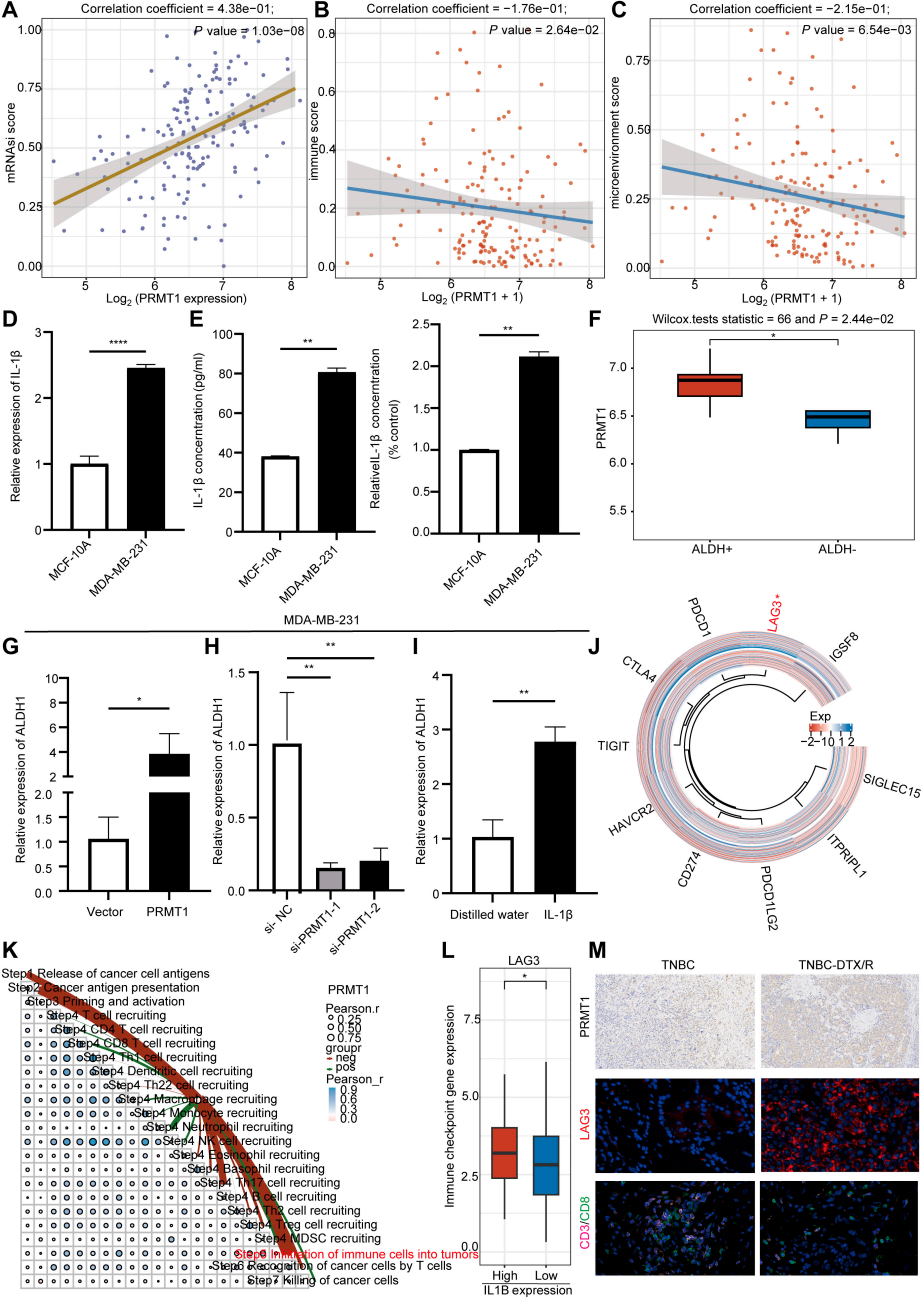
PRMT1 enhances tumor stemness and immune suppression in TNBC, sustaining chemoresistance. (A) The “stemness” score of PRMT1 was assessed. (B and C) The XCELL immune scores were employed to investigate the association between PRMT1 and the tumor microenvironment in TNBC. (D and E) The expression levels of IL-1β in MDA-MB-231 and MCF-10A cell lines were quantified through RT-qPCR and ELISA assays. (F) The expression of PRMT1 in ALDH^+^ and ALDH^−^ cells was compared in the dataset GSE136287. (G and H) RT-qPCR was conducted to evaluate the expression of ALDH1 following the knockdown and overexpression of PRMT1 in MDA-MB-231 cells. (I) The expression of ALDH1 in MDA-MB-231 cells was assessed via RT-qPCR subsequent to the administration of recombinant IL-1β protein. (J) A heatmap analysis was conducted to explore the relationship between PRMT1 and immune checkpoint. (K) The tumor immunophenotype analysis was performed to evaluate the proportion of tumor-infiltrating immune cells within the cancer immune cycle in relation to PRMT1 expression. (L) A boxplot analysis was executed to investigate the association between IL1B and LAG3. (M) Immunohistochemistry and immunofluorescence assay were used to examine the expression of PRMT1, LAG3 CD3, and CD8 in DTX/R or control TNBC tissues.

Enhanced tumor stemness and immune suppression are critical factors contributing to chemoresistance in breast cancer [53,54]. The tumor stemness is negatively correlated with the abundance of the immune microenvironment [55]. Our analysis, utilizing the “stemness” score, revealed a strong correlation between elevated PRMT1 expression and increased “stemness” score (Fig. 7A). Moreover, utilizing the XCELL immune scores and the tumor immune estimation resource (TIMER) immune scores, we discovered a negative correlation between PRMT1 expression and both immune scores and immune microenvironment scores (Fig. 7B and C and Fig. S7A). IL-1β is linked to tumor stemness and the immune microenvironment in breast cancer [56,57]. This suggests that PRMT1 may regulate IL-1β expression, thereby promoting tumor stemness and immune suppression in TNBC, which in turn sustains chemoresistance. Utilizing real-time quantitative polymerase chain reaction (RT-qPCR) and enzyme-linked immunosorbent assay (ELISA) assays, we observed that TNBC cells exhibited elevated secretion of IL-1β (Fig. 7D and E and Fig. S7D and E). ALDH1 is recognized as a pivotal marker of breast cancer stem cells [58]. An analysis of the GSE90564 dataset, which comprises chemoresistant tissues from TNBC, highlighted a positive correlation between the expression of PRMT1 and ALDH1 (Fig. S7B). In addition, PRMT1 expression was higher in ALDH^+^ breast cancer cells (Fig. 7F). The overexpression of PRMT1 led to an increase in ALDH1 expression in TNBC (Fig. 7G and Fig. S7G). Conversely, the knockdown of PRMT1 resulted in a reduction of ALDH1 expression (Fig. 7H and Fig. S7H). Analysis of the TCGA database shows a positive correlation between IL-1β and ALDH1 (Fig. S7F). Treatment of TNBC with recombinant IL-1β protein resulted in the up-regulation of ALDH1 expression (Fig. 7I and Fig. S7I). High PRMT1 expression is linked to improved immunotherapy response (Fig. S7J). Furthermore, PRMT1 and IL1B are linked to increased LAG3 expression (Fig. 7J and L). The tumor immunophenotype analysis indicates that elevated PRMT1 levels hinder immune cell infiltration in tumors (Fig. 7K). In DTX/R TNBC tissues, both PRMT1 and LAG3 are highly expressed, while CD3 and CD8 are lowly expressed (Fig. 7M), suggesting that in TNBC, PRMT1 boosts LAG3 expression, causing immune suppression and chemoresistance.

## Discussion

As introduced, TNBC remains a clinical challenge due to its high metastatic potential, dismal prognosis, and limited therapeutic options. The scarcity of targeted therapies and frequent development of chemoresistance underscore the urgent need to unravel the molecular mechanisms driving TNBC progression. Our initial aim was to clarify the role of PRMT1—a protein arginine methyltransferase previously linked to tumor proliferation—in mediating lung metastasis and chemoresistance, given its uncharacterized function in these contexts.

**Fig. 8.**
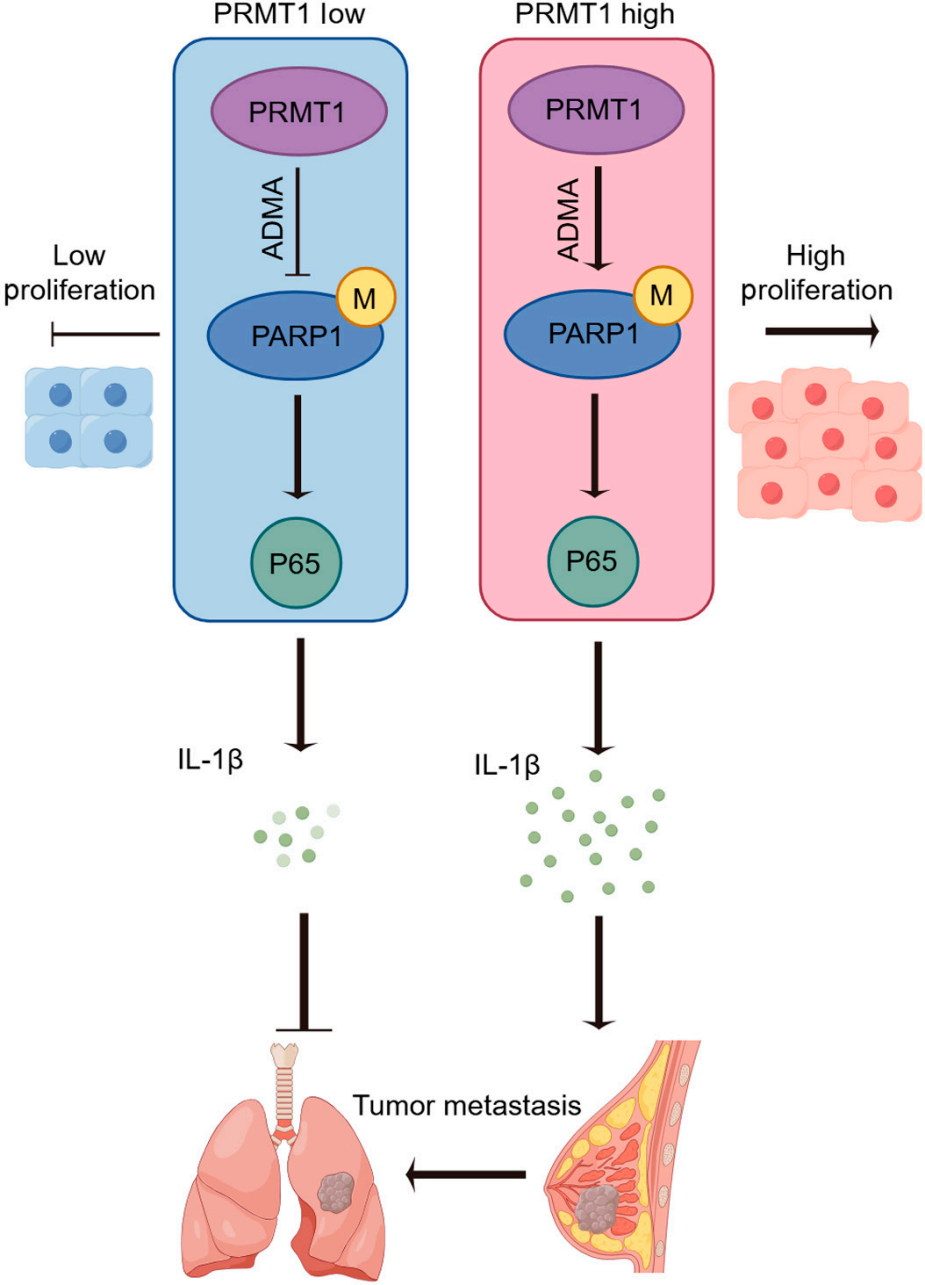
PRMT1-mediated PARP1 methylation drives lung metastasis and chemoresistance via P65 activation in TNBC.

We aim to clarify the complex role of PRMT1 in TNBC, an aggressive and treatment-resistant breast cancer subtype, by exploring its impact on cell proliferation, migration, invasion, chemoresistance, and lung metastasis. Our initial findings show that PRMT1 overexpression significantly enhances TNBC cell proliferation, underscoring its role in promoting tumor growth. Conversely, reducing PRMT1 expression markedly decreases cell proliferation, highlighting its critical role in TNBC cell growth. We showed that PRMT1 is crucial for enhancing the migration and invasion of TNBC cells, key factors in tumor spread and metastasis. Cells overexpressing PRMT1 exhibited increased migratory and invasive capabilities, while PRMT1 knockdown significantly reduced these traits, highlighting its role in TNBC’s aggressive behavior. Furthermore, we explored the impact of PRMT1 on chemoresistance, which is a persistent obstacle in the treatment of TNBC. Chemoresistance often leads to treatment failure and poor prognosis in TNBC patients. Docetaxel treatment assays revealed that PRMT1 overexpression confers chemoresistance, while silencing PRMT1 restores drug sensitivity. This underscores the potential of targeting PRMT1 as a means to overcome chemoresistance in TNBC, offering hope for improving treatment outcomes in patients with this difficult-to-treat cancer subtype. In addition to its role in migration, invasion, cell proliferation, and chemoresistance, PRMT1’s role in lung metastasis was thoroughly examined using a mouse model of TNBC. The findings revealed that PRMT1 overexpression significantly accelerated the formation of metastatic lesions in the lungs, while PRMT1 knockdown notably inhibited this process. This suggests that PRMT1 not only facilitates the primary tumor’s aggressiveness but also plays a pivotal role in metastatic spread, particularly to distant organs such as the lungs. Collectively, this research offers valuable insights into the mechanisms by which PRMT1 contributes to the progression and malignancy of TNBC. The ability of PRMT1 to modulate critical processes such as cell cycle regulation, migration, invasion, drug resistance, and metastasis underscores its significance as a potential therapeutic target (Fig. 8).

Despite the promising findings, the study is not without its limitations. The precise molecular mechanisms through which PRMT1 regulates these processes remain incompletely understood, and further research is needed to investigate its interactions within various signaling pathways. Additionally, while the in vitro and animal model studies provide strong evidence for PRMT1’s role in TNBC, clinical validation is necessary to assess the therapeutic potential of targeting PRMT1 in human patients. Thus, future research should focus on optimizing targeted PRMT1 therapies, determining their clinical effectiveness, and advancing these strategies from preclinical models to clinical trials, with the ultimate goal of improving patient outcomes in TNBC.

The therapeutic implications of targeting PRMT1 are vast and promising. By forming a stable interaction with PRMT1, TC-E 5003 demonstrates its ability to disrupt PRMT1’s function, thereby impairing the biological processes necessary for cancer cell survival and metastasis. The specificity of TC-E 5003 for PRMT1 suggests that further refinements of the molecule could lead to the development of a highly selective and potent inhibitor that can minimize off-target effects and reduce toxicity. In addition, combining PRMT1 inhibitors with existing therapies like chemotherapy may address one of the most significant challenges in TNBC treatment: chemoresistance. TNBC is notoriously aggressive and resistant to many forms of conventional treatment, necessitating the development of innovative approaches such as combination therapy. By sensitizing tumor cells to chemotherapy, PRMT1 inhibitors could enhance the effectiveness of current treatments, improving patient outcomes and prolonging survival. PRMT1’s influence on the immune microenvironment suggests new possibilities for immunotherapy. Combining PRMT1 inhibition with immune checkpoint inhibitors like PD-1/PD-L1 could strengthen the immune response against TNBC. PRMT1 may play a key role in tumor escape from immune detection, and its inhibition could reactivate the immune system against the tumor. In conclusion, PRMT1 inhibitors have the potential to revolutionize the treatment of TNBC, particularly for patients with limited treatment options. Further investigation into the precise mechanisms by which PRMT1 drives cancer progression, as well as its interaction with other therapeutic agents, will be crucial for optimizing treatment strategies. As our understanding of PRMT1’s role in TNBC deepens, it may become a central target in combating this aggressive disease.

The clinical significance of PRMT1 as a potential therapeutic target in TNBC is underscored by its pivotal role in driving metastasis and chemoresistance. The development of PRMT1-specific inhibitors, such as TC-E 5003, holds promise as a novel therapeutic strategy. Clinical trials evaluating PRMT1 inhibitors in TNBC could focus on 2 key objectives: (a) monotherapy to assess antitumor efficacy in patients with high PRMT1 expression and (b) combination therapy with docetaxel or immune checkpoint inhibitors to overcome chemoresistance and enhance immune surveillance. Notably, PRMT1’s role in modulating tumor stemness and immune suppression suggests that its inhibition may synergize with immunotherapies by reactivating antitumor immune responses. Ultimately, integrating PRMT1 inhibition into combination regimens may offer a precision medicine approach to tackle the heterogeneity and therapeutic resistance that characterize TNBC.

## Materials and Methods

### Cell culture

Two TNBC cell lines (MDA-MB-231 and BT-549) and a normal breast epithelial cell line (MCF-10A) were used, all sourced from our laboratory’s cell bank. MDA-MB-231 and BT-549 were cultured in high-glucose Dulbecco’s modified Eagle’s medium (Gibco, USA) with 10% fetal bovine serum (FBS) (Excell Bio, FSD500) at 37 °C and 5% CO_2_. MCF-10A was maintained in a specialized growth medium (Pricella, #CL-0525).

### RNA extraction and real-time RT-PCR

RNA was completely extracted from the cultured cells utilizing the Fast Cell Total RNA Extraction Kit (ES Science, RN001), which is designed for efficient RNA isolation. Complementary DNA (cDNA) synthesis was performed using the Premix PrimeScript RT Master Mix (Takara, RR036A) in accordance with the manufacturer’s instructions. For RT-qPCR analysis, TB Green Premix Ex Taq (Takara, RR420A) was employed to amplify specific gene sequences, enabling precise quantification of gene expression levels.

### Western blot

Cells were lysed in a buffer (Beyotime, P0013B) with phenylmethylsulfonyl fluoride (Beyotime, ST506) to extract proteins, which were quantified using a bicinchoninic acid (BCA) protein assay (GLPBIO, GK10009). Equal protein amounts were separated by sodium dodecyl sulfate–polyacrylamide gel electrophoresis (SDS-PAGE) and transferred to a polyvinylidene difluoride membrane (Millipore, IPVH00010). The membrane was blocked with 5% nonfat milk (BBI Life Science, #F109BA0020) and incubated with primary and secondary antibodies, and protein bands were visualized using an enhanced chemiluminescence detection system. The antibodies used are as follows: PRMT1 (Proteintech, 11279-1-AP), PARP1 (Proteintech, 13371-1-AP), P65 (Proteintech, 10745-1-AP), and β-tubulin (Proteintech, 10094-1-AP).

### Gene transfection

Overexpression vectors were introduced into the cells utilizing the P3000 Reagent (Invitrogen, L3000001) in conjunction with Lipofectamine 3000. For the transfection of siRNA, Lipofectamine 3000 Reagent was used.

### Colony formation and CCK-8 assay

In the colony formation assay, 2,000 cells were seeded per well in a 6-well plate and conducted in triplicate. After 3 weeks, colonies were fixed with paraformaldehyde and stained with crystal violet (Solarbio, C8470). For the CCK-8 assay, 3,000 cells were seeded per well in a 96-well plate, with 5 replicates per condition, and cell proliferation was measured using the CCK-8 Kit (GLPBio, GK10001).

### Transwell migration and wound healing assay

In the Transwell migration assay, cells were placed in the upper chamber, with the lower chamber containing 500 μl of medium with 20% FBS. After 24 h, cells were fixed with paraformaldehyde and stained with crystal violet. For the wound healing assay, cells in a 6-well plate were scratched with a pipette tip, and images were taken at 0 and 24 h to assess wound closure.

### Cell cycle and apoptosis analysis

A total of 1 × 10^6^ cells were collected for cell cycle analysis and subjected to fixation overnight with 3 ml of pre-chilled anhydrous ethanol at −20 °C. Post-fixation, the cells were stained with propidium iodide (PI) (Multi Sciences Biotech Co., CCS012) and subsequently analyzed via flow cytometry to ascertain the distribution of cells across various phases of the cell cycle. In the context of apoptosis analysis, 1 × 10^6^ cells were collected and resuspended in 500 μl of 1× Binding Buffer. To this suspension, 5 μl of Annexin V–fluorescein isothiocyanate and 10 μl of PI (Multi Sciences Biotech Co, AT101) were added. The cells were then incubated at room temperature in the dark for 5 min to facilitate binding. Following incubation, apoptotic and necrotic events were assessed using flow cytometry, thereby providing a comprehensive evaluation of cell death pathways.

### Immunoprecipitation

The lysates were incubated overnight at 4 °C with a commercial antibody (1 to 4 μg/ml) targeting the proteins of interest, followed by the addition of protein A/G magnetic beads (MCE, HY-K0202-1) to capture the antibody–protein complexes. These complexes underwent 3 washes with phosphate-buffered saline with Tween 20 (PBST) to eliminate nonspecific interactions. The immune complexes were then resuspended in 1× SDS-PAGE loading buffer (Beyotime, P0015A) for subsequent analysis. Western blot analysis was employed to detect the bound proteins. For MS analysis, the magnetic beads were removed, and the proteins were submitted to Biotechnology Co. Ltd. (Guangzhou, China) for comprehensive protein identification.

### Silver staining

Upon completion of protein electrophoresis, the gel underwent a meticulous fixation process and was subsequently washed with a 30% ethanol solution, followed by a rinse with distilled water to eliminate any residual reagents. The gel was then treated with a rapid silver staining kit (Beyotime, P0017S) to facilitate the visualization of the resolved proteins. Post-staining, images of the gel were captured to enable subsequent analysis, thereby offering a clear and precise visual depiction of the protein bands.

### Immunofluorescence

Cells were cultured in a 24-well plate, prepared as slides, and fixed with paraformaldehyde. They were then permeabilized with 0.3% Triton X-100 (Beyotime, P0096) in PBS for 15 min and blocked with 5% bovine serum albumin (BSA) (Solarbio, A8020) in PBS for 1 h to prevent nonspecific binding. The slides were incubated with primary antibodies, followed by fluorescently labeled secondary antibodies. Images were captured using a laser confocal microscope to observe protein localization and expression.

### Enzyme-linked immunosorbent assay

Cell culture supernatants were initially collected and subjected to centrifugation at 300*g* for 10 min to eliminate cellular debris. The supernatants were then appropriately diluted and analyzed utilizing the Human IL-1β ELISA Kit (Multi Sciences Biotech Co., EK101B). Following the color development phase, absorbance was measured at a wavelength of 450 nm, which corresponds to the peak absorption. The concentration of IL-1β in the samples was determined by comparing the measured absorbance values against a standard curve, facilitating precise quantification.

### Xenograft tumor in vivo

For the in vivo tumor studies, 4-week-old female BALB/c nude mice were used. To create subcutaneous tumors, 1 × 10^7^ MDA-MB-231 cells in 100 μl of PBS were injected into each mouse’s flank. Tumor growth was regularly monitored, and volume was calculated using (length × width^2^)/2, starting 2 weeks after injection. For lung metastasis, 2 × 10^6^ MDA-MB-231 cells were injected into the tail vein, and lungs were collected a month later for analysis. All procedures were approved by the Animal Care and Use Committee of Sun Yat-sen University, adhering to ethical guidelines.

### H&E staining

Tumor tissues were collected for histological analysis, fixed for 24 h, dehydrated, embedded in paraffin, and sliced thinly. The sections were deparaffinized in xylene, rehydrated with decreasing ethanol concentrations, and stained with H&E. After staining, they were dehydrated again in xylene, mounted with neutral resin, and examined microscopically.

### Immunohistochemistry

Breast cancer tissue samples were collected from SYSUCC in Guangzhou, China, following ethical guidelines. Antigen retrieval used EDTA citrate buffer (ZSGB-BIO, ZLI-9069) in a pressure cooker. Tissues were blocked and incubated with primary and secondary antibodies and then washed with PBST. 3,3′-Diaminobenzidine (DAB) (ZSGB-BIO, ZLI-9017) staining visualized antibody binding, and hematoxylin counterstaining highlighted the nuclei. The antibodies used are as follows: PRMT1 (Proteintech, 11279-1-AP) and Ki67 (Proteintech, 27309-1-AP).

### Statistical analysis

Data analysis was performed using GraphPad Prism 9. A Student’s *t* test assessed statistical significance between experimental groups, while the Spearman correlation coefficient evaluated potential relationships or trends.

## Data Availability

The datasets used and/or analyzed in this study are reported in the article, and/or additional files are available from the corresponding authors.
