# Supplementary figures and images for: PRMT1-Mediated PARP1 Methylation Drives Lung Metastasis and Chemoresistance via P65 Activation in Triple-Negative Breast Cancer
Source: Research (Wash D C). 2025 Sep 8;8:0854. doi: 10.34133/research.0854 (PMC12415337; doi:10.34133/research.0854)

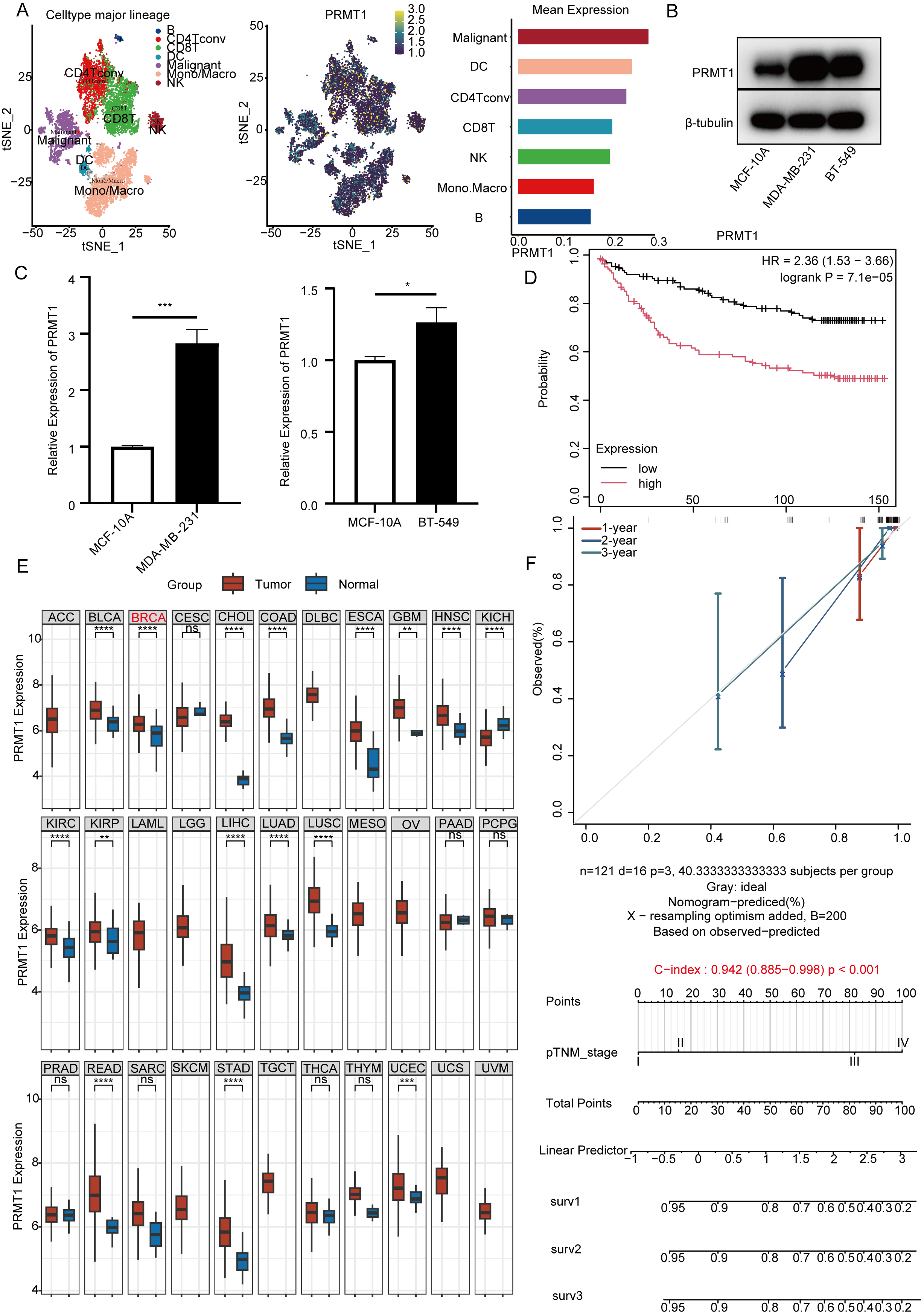

Supplement: Supplementary 1 — Figs. S1 to S7 Tables S1 to S4 [file research.0854.f1.zip › FigS1.tif]

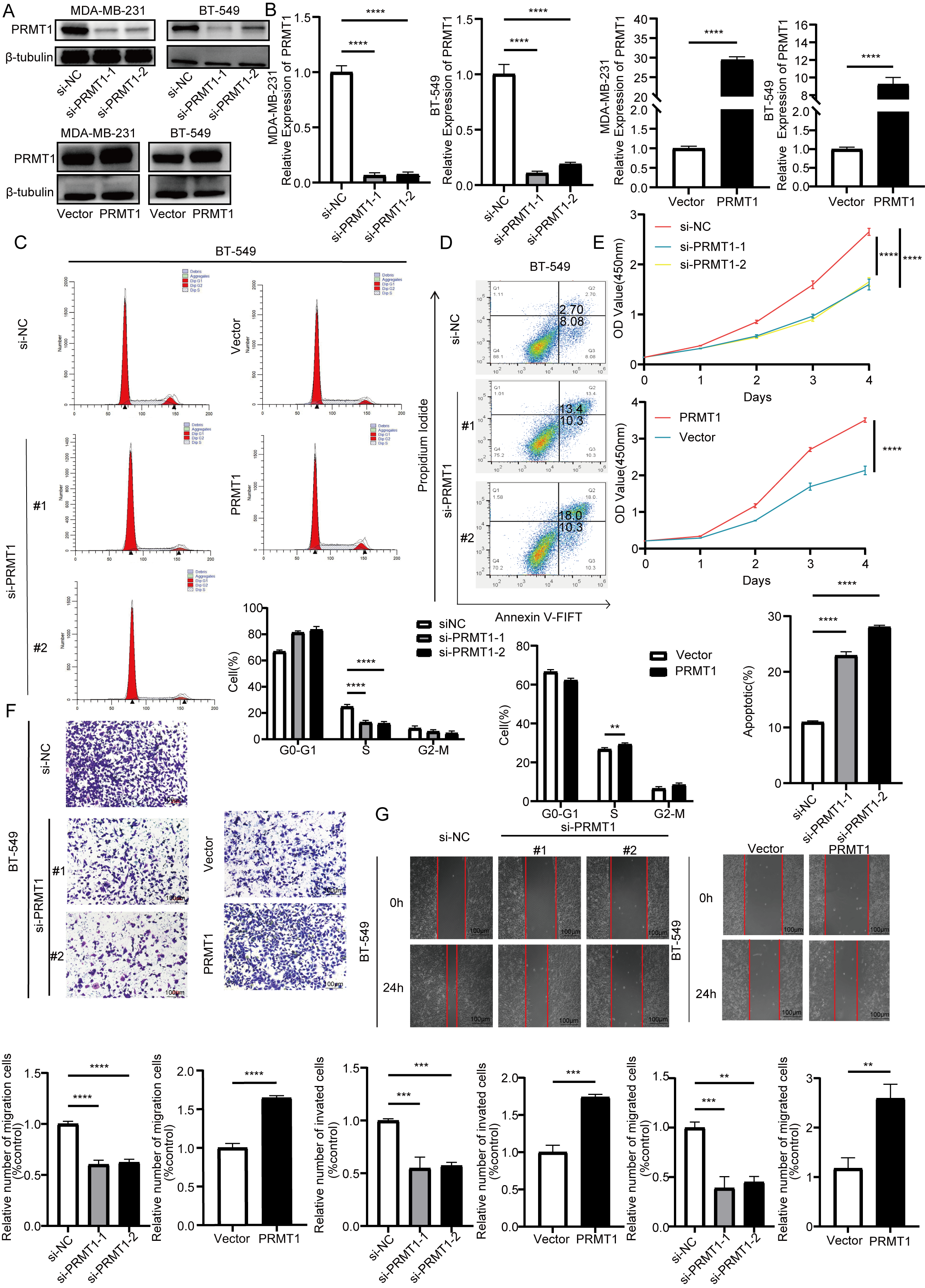

Supplement: Supplementary 1 — Figs. S1 to S7 Tables S1 to S4 [file research.0854.f1.zip › FigS2.tif]

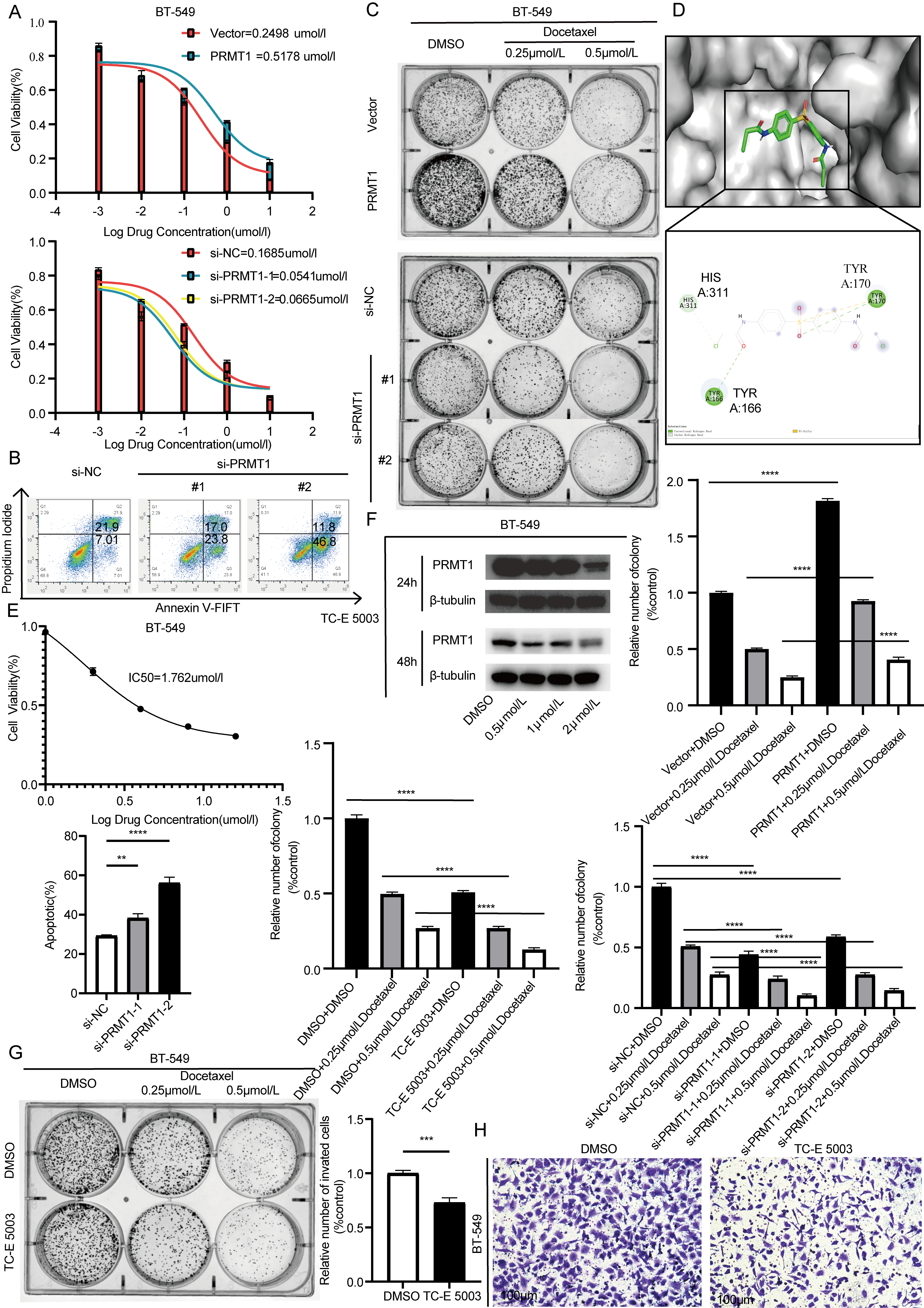

Supplement: Supplementary 1 — Figs. S1 to S7 Tables S1 to S4 [file research.0854.f1.zip › FigS3.tif]

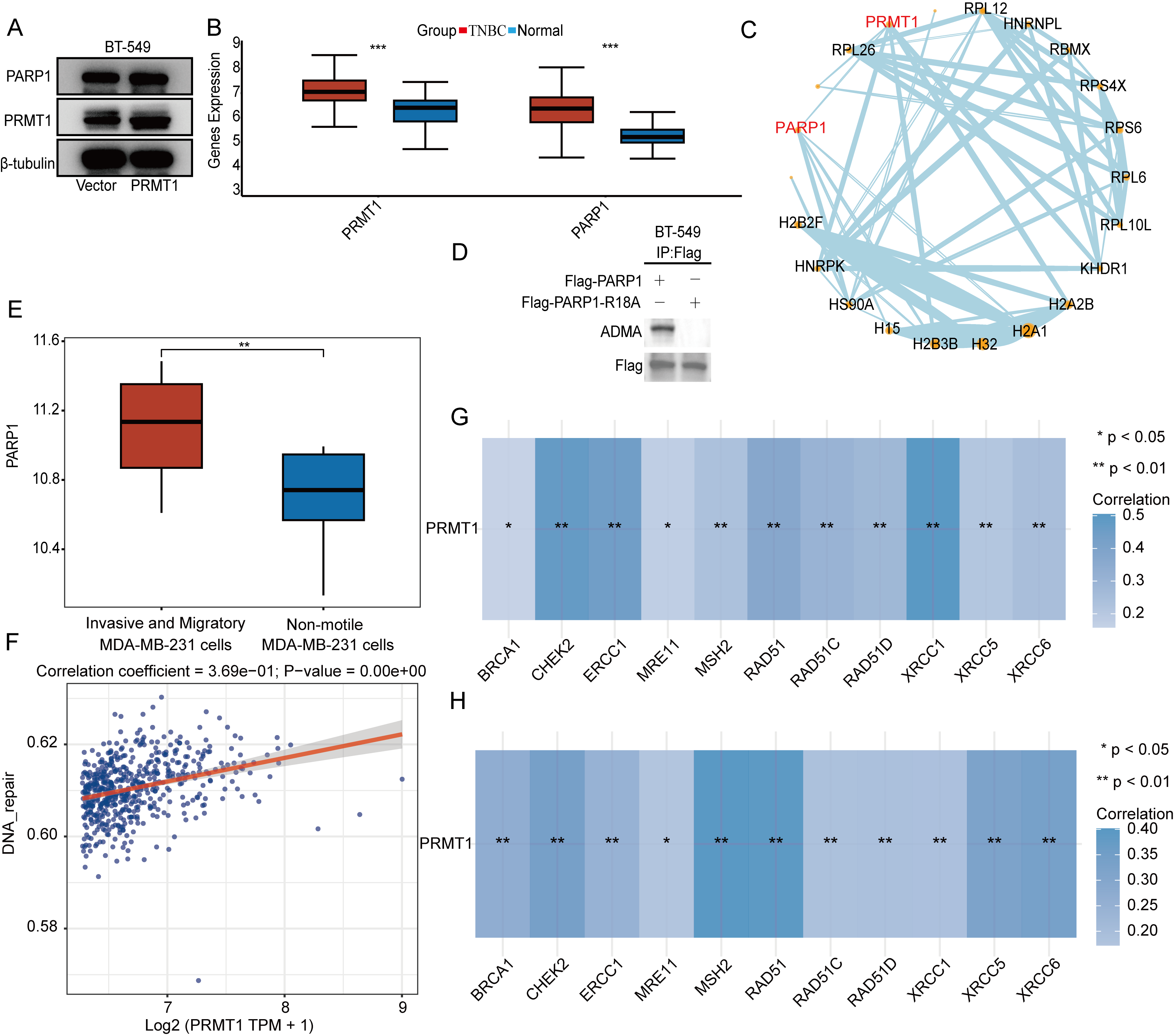

Supplement: Supplementary 1 — Figs. S1 to S7 Tables S1 to S4 [file research.0854.f1.zip › FigS4.tif]

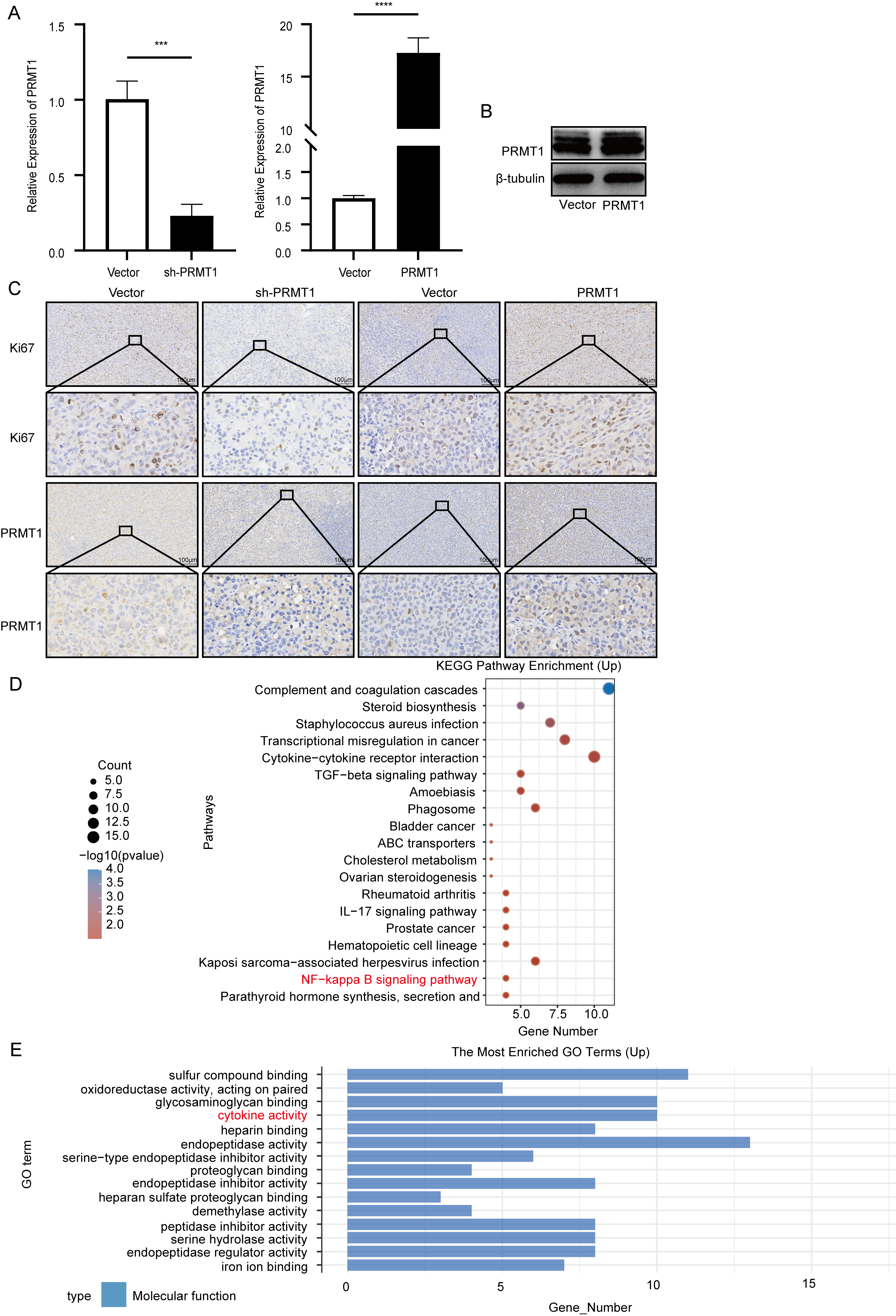

Supplement: Supplementary 1 — Figs. S1 to S7 Tables S1 to S4 [file research.0854.f1.zip › FigS5.tif]

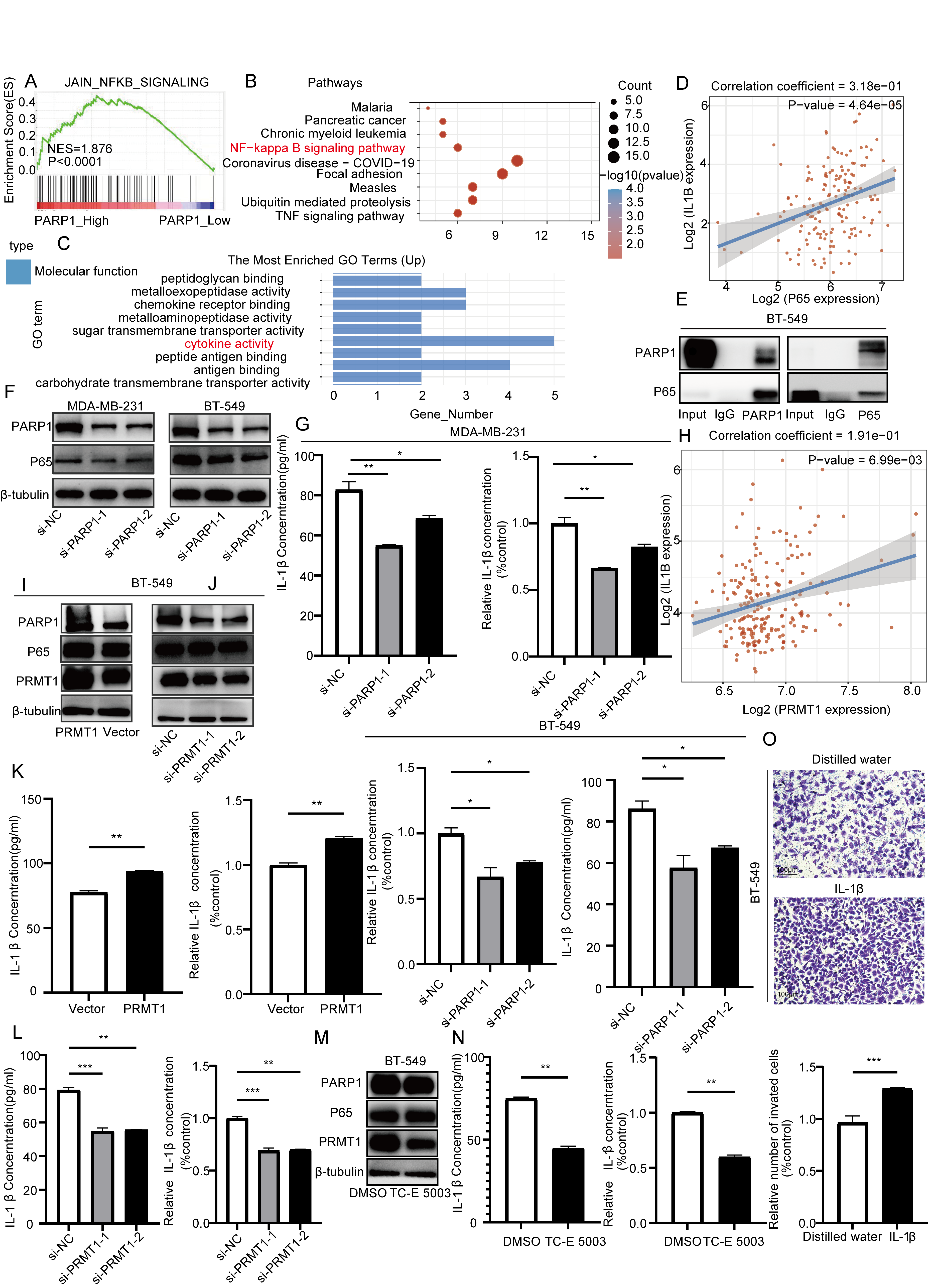

Supplement: Supplementary 1 — Figs. S1 to S7 Tables S1 to S4 [file research.0854.f1.zip › FigS6.tif]

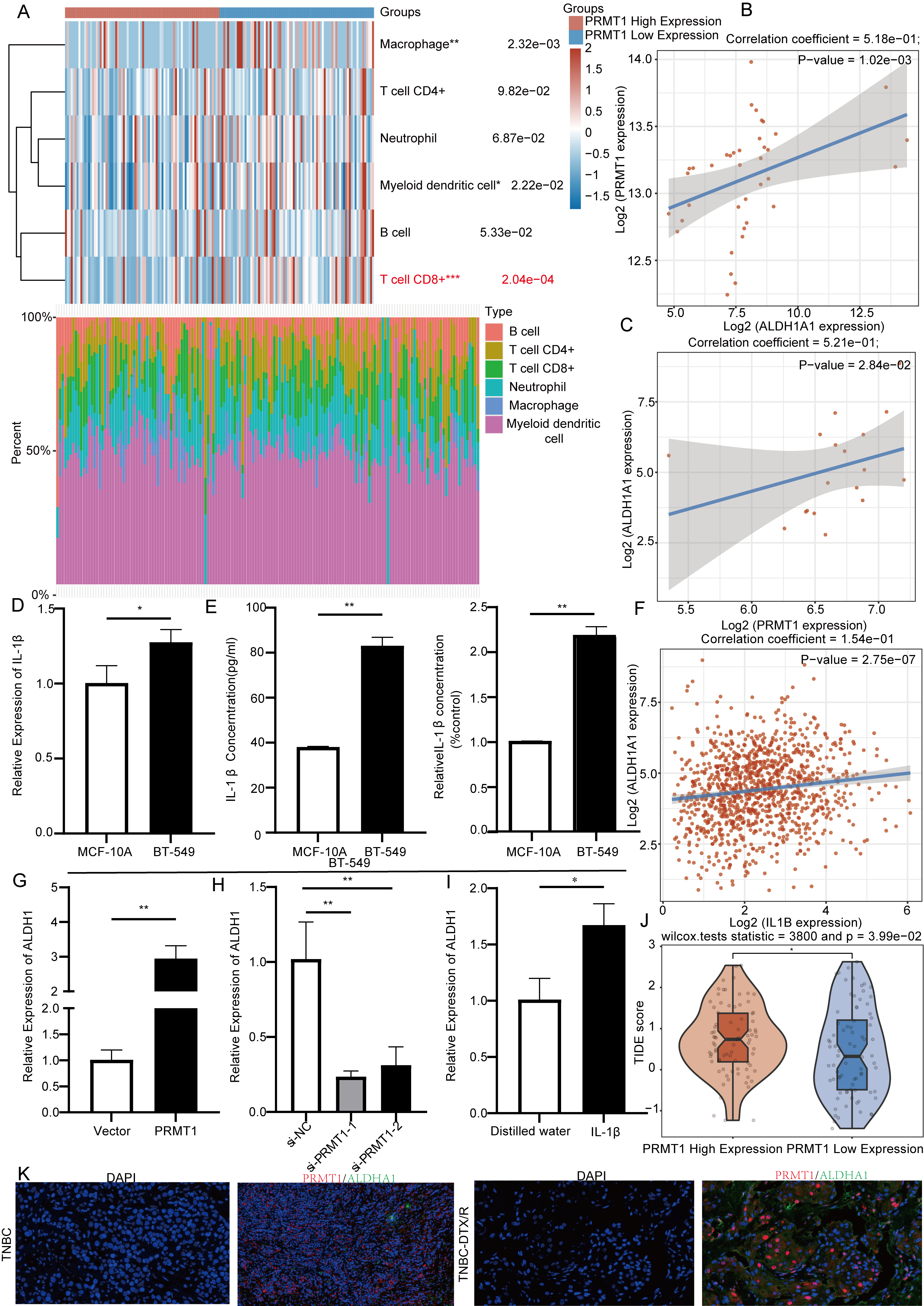

Supplement: Supplementary 1 — Figs. S1 to S7 Tables S1 to S4 [file research.0854.f1.zip › FigS7.tif]

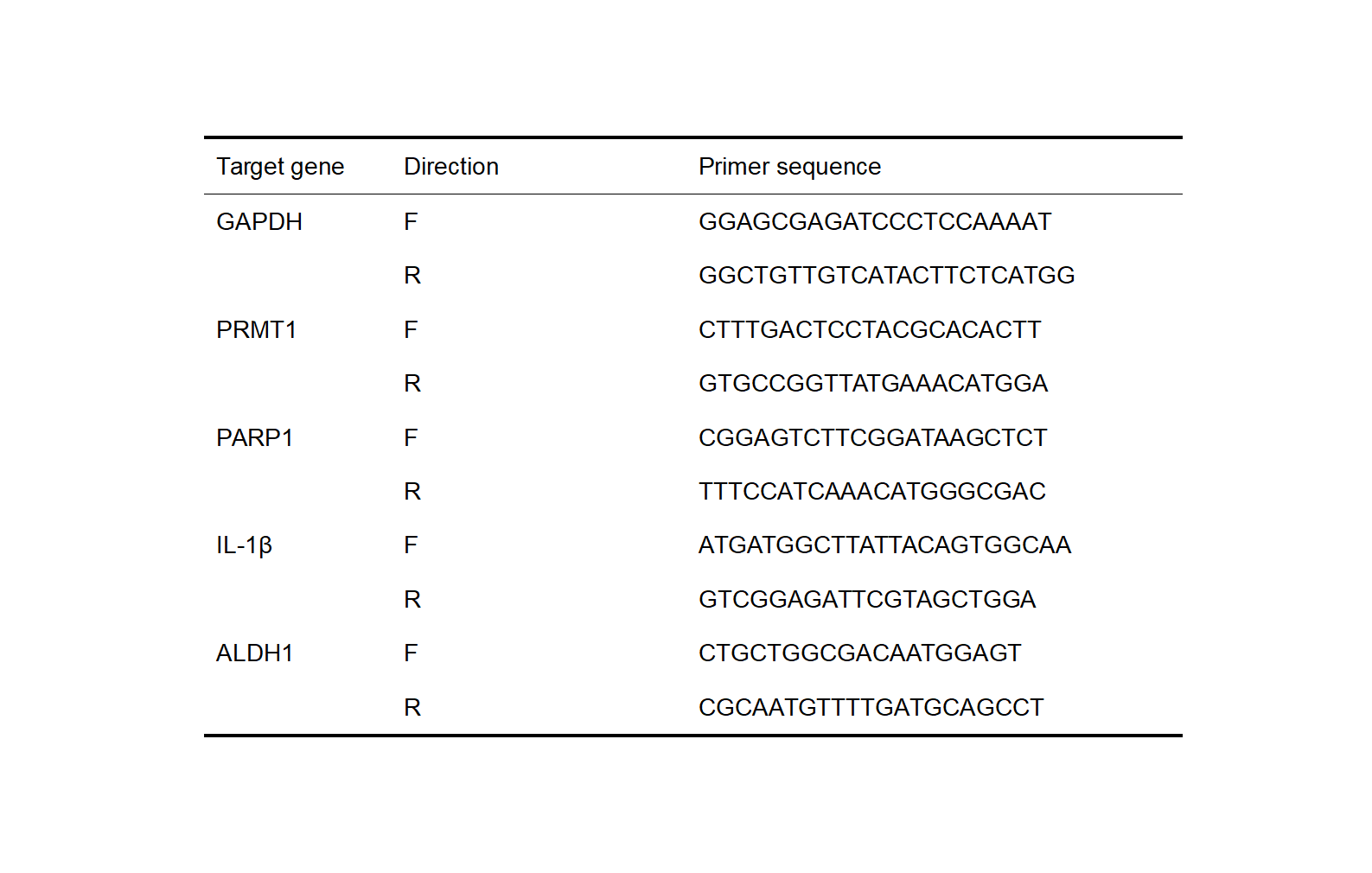

Supplement: Supplementary 1 — Figs. S1 to S7 Tables S1 to S4 [file research.0854.f1.zip › Supplementary Table S1. Primer sequence.tif]

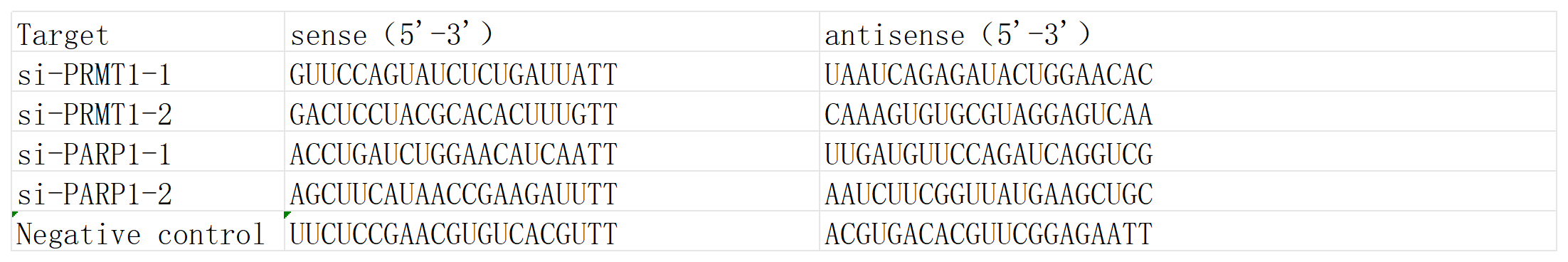

Supplement: Supplementary 1 — Figs. S1 to S7 Tables S1 to S4 [file research.0854.f1.zip › Supplementary Table S2. siRNAs sequence.tif]

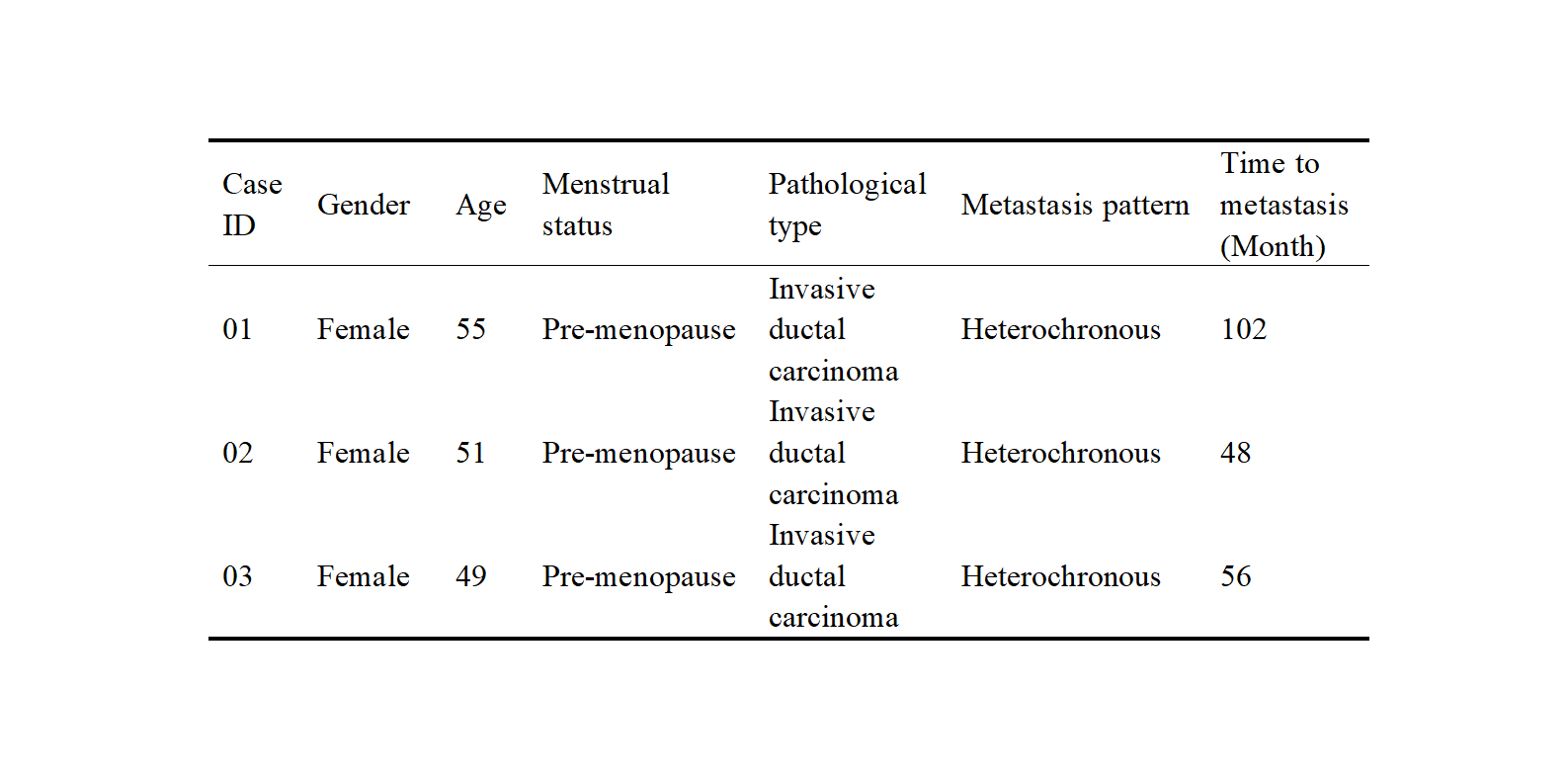

Supplement: Supplementary 1 — Figs. S1 to S7 Tables S1 to S4 [file research.0854.f1.zip › Supplementary Table S3.tif]

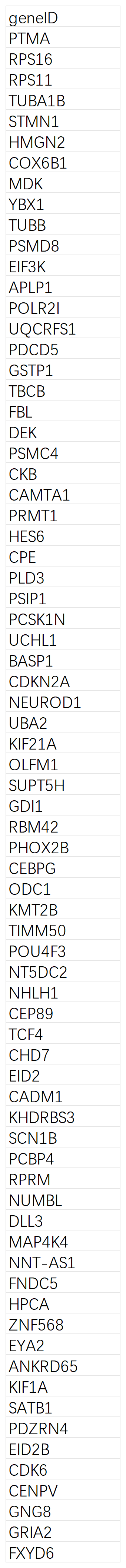

Supplement: Supplementary 1 — Figs. S1 to S7 Tables S1 to S4 [file research.0854.f1.zip › Supplementary Table S4. Cell cycle related gene sets by scRNA-seq in this study.tif]
